# Factors associated with antibiotic use in children hospitalized for acute viral gastroenteritis and the relation to rotavirus vaccination

**DOI:** 10.1080/21645515.2024.2396707

**Published:** 2024-09-09

**Authors:** Muna Omar, Eias Kassem, Emilia Anis, Roula Abu-Jabal, Basher Mwassi, Lester Shulman, Dani Cohen, Khitam Muhsen

**Affiliations:** a Department of Epidemiology and Preventive Medicine, School of Public Health, Faculty of Medical and Health Sciences, Tel Aviv University, Tel Aviv, Israel; b Department of Pediatrics, Hillel Yaffe Medical Center, Hadera, Israel; c Division of Epidemiology, Ministry of Health, Jerusalem, Israel; d Central Virology Laboratory, Ministry of Health, Ramat Gan, Israel

**Keywords:** Viral gastroenteritis, rotavirus, rotavirus vaccine, antibiotic use, children

## Abstract

Evidence on unnecessary antibiotic use in children with acute viral gastroenteritis (AGE) is scarce. We characterized the extent and correlates of antibiotic use among children hospitalized with viral AGE. A single-center study enrolled children aged 0–59 months hospitalized for AGE between 2008 and 2015 in Israel. Information was collected on laboratory tests, diagnoses, antibiotic treatment, and rotavirus vaccination. Stool samples were tested for rotavirus antigen, GII-norovirus, and stool cultures were performed for bacterial enteropathogens. Data from 2240 children were analyzed. Rotavirus vaccine was given to 79% of eligible children. Rotavirus test was performed on 1419 (63.3%) children. Before the introduction of universal rotavirus vaccination (2008–2010), rotavirus positivity in stool samples was 37.0%, which declined to 17.3% during the universal vaccination years (2011–2015). Overall, 1395 participants had viral AGE. Of those, 253 (18.1% [95% CI 16.1–20.2]) had unnecessary antibiotic treatment, mostly penicillin 46.6%, ceftriaxone 34.0% and azithromycin 21.7%. A multivariable analysis showed an inverse association between rotavirus vaccination and unnecessary antibiotic treatment (odds ratio = 0.53 [95% CI 0.31–0.91]), while positive associations were found with performing chest-X-ray test (3.00 [1.73–5.23]), blood (3.29 [95% CI 1.85–5.86]) and urine cultures (7.12 [3.77–13.43]), levels of C-reactive protein (1.02 [1.01–1.02]) and leukocytes (1.05 [1.01–1.09]). The results were consistent in an analysis of children with laboratory-confirmed rotavirus or norovirus AGE, or after excluding children with CRP > 50 mg/L. In conclusion, antibiotic prescription was common among hospitalized children with viral AGE, which was inversely related to rotavirus vaccination, possibly due to less severe illness in the vaccinated children.

## Introduction

Acute gastroenteritis (AGE) is a major cause of morbidity and mortality in children, with most deaths occurring in low-middle-income countries.^1,2^ AGE is mainly caused by viral pathogens^1–3^ with rotavirus being the leading cause of severe AGE in young children.^3–6^ The main recommended treatment for AGE is a fluid replacement for hydration.^7–9^ Antibiotic treatment is recommended only for patients with dysentery or certain bacterial infections such as shigellosis, severe salmonellosis or cholera.^7,10^

Several studies, predominantly conducted in low-middle-income countries, have shown high antibiotic use ranging from 20.0% to 86.9% in children with AGE, even in children with viral AGE.^5,11–14^ The Global Enteric Multicenter Study (GEMS) conducted in seven countries in sub-Saharan Africa and South Asia showed that rotavirus AGE (RVGE) was the leading clinically-attended, antibiotic-treated diarrhea in children aged less than two years, constituting 29.2% of antibiotic-treated cases.^5^ Another multicenter study conducted in eight sites in Africa, Asia, and South America demonstrated a high incidence of antibiotic use for rotavirus diarrhea, 10.9 courses per 100 child-years.^11^

Vaccines might play a role in combatting antimicrobial resistance.^15–17^ Bacterial vaccines can directly prevent the occurrence of bacterial infections with resistant strains targeted by the vaccine^17^ and indirectly impact antimicrobial resistance by reducing antibiotic use,^18,19^ as demonstrated for *Haemophilus influenzae type b* and pneumococcal conjugate vaccines.^19,20^ Viral vaccines might reduce antibiotic use by reducing disease incidence,^21,22^ as was shown for influenza vaccines.^22,23^

Several oral live attenuated rotavirus vaccines are currently available, including the pentavalent rotavirus vaccine (RotaTeq™, Merck & Co., Whitehouse, Pennsylvania),^24,25^ the monovalent vaccine (Rotarix™, GSK Biologicals, Rixensart)™,^26–28^ Rotavac™ (Bharat Biotech International Ltd., Hyderabad, India) and pentavalent vaccine Rotasiil™ (Serum Institute of India, Pune, India).^27–31^ Rotavirus vaccines have been introduced to the national immunization programs of over 100 countries^32^ and led to a substantial reduction in the burden of rotavirus and all-cause AGE.^33–38^

Evidence on antibiotic use in children with AGE in high-income country settings and on the role of rotavirus vaccines in antibiotic use remains elusive. A modeling study^15^ showed an 8.1% lower odds of antibiotic-treated diarrhea in relation to vaccination with the full series of rotavirus vaccines in children from low-middle-income countries.^15^ A study from the United States based on real-world data showed a ~ 21% reduction in the risk of antibiotic prescription following a diagnosis of AGE in children with full rotavirus vaccination versus unvaccinated ones.^39^ Given the paucity of real-world evidence on the extent and correlates of unnecessary antibiotic use in children with viral AGE from high-income countries, as well as on the role of rotavirus vaccination in antibiotic use in these settings, we aimed to address these gaps. The specific aims of the study were to characterize antibiotic prescriptions among children aged 0–59 months hospitalized for viral AGE and examine factors associated with unnecessary antibiotic use, including the role of rotavirus vaccination, in Israel, while capturing both the pre-and-post universal rotavirus vaccination eras. Our hypothesis was that antibiotic use in children with viral AGE might be positively associated with indicators of severe illness and negatively associated with rotavirus vaccination.

## Materials and methods

A retrospective study was undertaken using data from hospital-based surveillance of AGE and RVGE hospitalizations between November 2007 and December 2015 in Israel, a high-income country and member of the OECD states.^40^ The study design was described before.^41^ Briefly, the study was conducted at the pediatric department in Hillel Yaffe Medical Center, a 500-bed hospital that mainly serves the population of the Hadera sub-district. During the study period, nearly 400 thousand residents lived in this region; of those 35,700 -39,800 were under the age of five years.^42,43^ Access to healthcare services in Israel is universal, based on the National Health Insurance Law.^44^ In-patient care is provided by public hospitals. Childhood vaccines included in the national immunization program are given at the Maternal and Child Health Clinics at no cost to parents.^45^ Both RotaTeq™ and Rotarix™ were licensed in Israel in 2007 and became available through Health Maintenance Organizations,^46^ with partial reimbursement for parents who were interested in vaccinating their children, both vaccines were used during 2007–2010 but with relatively low uptake.^47^ Universal vaccination using RotaTeq™ was introduced in Israel in December 2010; the vaccine is administered in three doses at ages two, four, and six months at no cost to parents. Since then, RotaTeq™ is the only rotavirus vaccine used in Israel, with 80% coverage of three doses.^47,48^ There are regulations on antibiotics dispensing in Israel, similar to other high-income countries^49,50^ which require prescriptions from a physician with a valid license to practice medicine.

The sampling frame included children 0–59 months of age hospitalized with diarrhea, defined as having three or more watery stools per 24 hours.

### Data collection and definition of the study variables

Data were collected prospectively in the framework of active hospital-based surveillance via interviews with the parents and from medical records on demographic factors and symptoms as previously described.^38,48,51,52^ Data on rotavirus immunization was obtained through linkage with the national immunization registry.^53,54^ Stool specimens were obtained from the participants within 24–48 hours of hospital admission and tested for rotavirus antigen and bacteria by stool culture at the admitting hospital. The stools were tested for norovirus and retested for rotavirus during the prospective phase of analysis after completing sample collection. In the retrospective component of the study, we collected new data on the same children and hospitalizations on antibiotic use, referral to laboratory tests, and the results of these tests. The retrospective data collection phase investigating factors related to antibiotic use between 2008 and 2015 was conducted between 2019 and 2021.^41^

### Classification of the infections

The dependent variable was unnecessary antibiotic use, defined as receiving antibiotic treatment in patients with viral AGE who did not have other reasons that justify antibiotic treatment as classified by two investigators: a senior pediatrician (EK) and a pharmacist/epidemiologist (MO).

Antibiotic treatment is justified if given to children with bacterial infections. Therefore, a major component of this study was to differentiate between bacterial and viral infections. The classification of bacterial and viral infections was undertaken by a senior pediatrician (EK) and a pharmacist/epidemiologist (MO), using all the available clinical and laboratory data in the medical records as previously described.^41^ Unlike physicians who in real-life clinical settings are required to order diagnostic tests and decide on treatment early upon the child’s admission to the hospital, before the test results become available (i.e., based on partial data), herein we retrospectively reviewed all the laboratory results and clinical data to define unnecessary antibiotic treatment. Accordingly, the definition of unnecessary antibiotic treatment was the prescription of antibiotics for children with viral AGE, lacking any other bacterial infection that justifies antibiotic treatment. Children with bacterial infections were identified and were excluded from the analysis of unnecessary antibiotic treatment. Information on the exact date of prescribing antibiotic treatment during hospitalization was lacking.

Bacterial AGE was defined on the basis of a positive stool culture for *Salmonella*, *Shigella*, or *Campylobacter*, or clinical dysentery, documented in the medical record by the presence of blood or mucus in the stool.^55,56^ The definition of bacterial co-infection was the presence of bacterial extraintestinal infection concurrently with AGE (Supplementary Table S1). Children who had blood leukocyte levels above 15 K/µl, neutrophil levels above 10 K/µl, and C-reactive protein (CRP) above 50 mg/L, who did not have a documented bacterial infection or bacterial AGE, were considered as likely to have a bacterial infection. Children with CRP >50 mg/L who had leukocyte ≤15 K/µl or neutrophil ≤10 K/µl were kept in the primary analysis but excluded in sensitivity analyses (see data analysis section).

The remaining children who did not have any of these infections were classified as having viral gastroenteritis, including children who tested positive for rotavirus antigen or norovirus by RT-PCR. RVGE was defined as hospitalization for diarrhea, with the detection of rotavirus antigen in a stool specimen.

Children with bacterial AGE, bacterial co-infections, or those classified as ‘likely having bacterial infection’ were excluded from the analysis on unnecessary antibiotic use in children with viral AGE. We followed this approach to minimize the potential misclassification of viral infections, and to increase the specificity of the definition of antibiotic misuse.

The independent variables were demographics (age, sex, ethnicity, residential socioeconomic status (SES)), laboratory results (including complete blood count (hemoglobin, platelets, leukocytes, neutrophils, lymphocytes), CRP, glucose, blood urea nitrogen (BUN), creatinine, sodium, and potassium which were analyzed as continuous variables), prior hospitalizations (yes or no), a prior AGE hospitalization except the current episode, background diseases (yes or no), symptoms (vomiting, bloody stool, number of stools in the severe day (0–5 and ≥ 6)), performing tests (urine, blood, or stool culture, or chest X-ray tests), receiving at least one dose of a rotavirus vaccine at least 14 days before hospital admission (yes or no), the calendar year of admissions, and birth cohort (before universal rotavirus vaccination (2008–2010) versus the universal rotavirus immunization years (2011–2015)), which all were analyzed as categorical variables.

### Laboratory methods

Stool specimens were collected from patients and promptly stored at temperatures between 2–8°C and transported to the hospital laboratory within a few hours after collection, where they were tested for the presence of rotavirus antigen by immunochromatography (Rotavirus Dipsticks, Hylabs Rehovot Israel) as per the manufacturer’s instruction. Standard microbiologic methods were employed to perform stool cultures of isolating *Salmonella*, *Shigella*, and *Campylobacter*. These tests were performed at the microbiology laboratory at Hillel Yaffe Medical Center, within 24–48 hours of hospital admission. The remaining fecal material was stored at −80°C and tested collectively using real-time RT-PCR for the detection of GII norovirus as previously described.^52,57–61^

### Statistical analysis

Descriptive statistics were employed using frequencies and percentages for categorical variables. Normally distributed continuous variables were expressed as means with standard deviation (SD), and skewed variables were presented as medians and interquartile range (IQR). Proportions of unnecessary antibiotic use among children with viral AGE were calculated per 100 children with a 95% confidence interval (CI), determined using the binomial-based mid-P method.^62^

The associations of sociodemographic and clinical characteristics, rotavirus vaccination referral to laboratory tests, and their results with unnecessary antibiotic use were examined using the chi-square test for categorical variables, the Student’s *t-*test for continuous variables, and the Mann-Whitney *U*-test for variables exhibiting a skewed distribution. A multivariable logistic regression model was applied to examine these associations while adjusting for other variables in the model. Variables with a *p* < .1 in the bivariate analysis were assessed for potential inclusion in the multivariable model. For each variable, odds ratio (OR) and 95% confidence interval (CI) were obtained from logistic regression models. A 2-tailed p-value <.05 was considered statistically significant. Multicollinearity between the independent variables was assessed using the variance inflation factor (VIF). The primary analysis included all children with viral AGE (e.g., excluding children with bacterial AGE, bacterial co-infection, and those with likely bacterial infection (i.e., CRP >50 mg/L and leukocyte level >15 K/µl, neutrophil levels >10 K/µl)). Sensitivity analyses were conducted while 1) excluding children with viral AGE who had a CRP level of >50 mg/L but their leukocyte level ≤15 K/µl, neutrophil levels ≤10 K/µl as another indication for bacterial involvement;^63^ 2) limiting the analysis for children eligible for universal rotavirus vaccination (birth cohorts 2011–2015, and again for the birth cohorts 2012–2015 while considering 2011 as a transitional year); 3) limiting the analysis for children with laboratory-confirmed RVGE or norovirus AGE, and 4) excluding from the analysis children who performed chest X-ray test, urine or blood cultures, as indications of potential systemic illness. The data were analyzed using IBM SPSS (IBM, Armonk, New York, NY, USA) software version 28 and Winpepi Software.^64^

#### Ethics approval

The study was conducted in accordance with the Declaration of Helsinki and approved by the Institutional Review Board (IRB) (Helsinki Committee) of Hillel Yaffe Medical Center and the Ministry of Health (protocol number 920,120,422). The current protocol was approved by the IRB of Hillel Yaffe Medical Center (Protocol number 0180–20-HYMC 20.12.2020) and the Ethics Committee of Tel Aviv University (protocol number 1–0003388 29.06.2021).

## Results

From November 2007 to October 2015, there were 2805 hospital admissions of children aged 0–59 months with AGE at Hillel Yaffe Medical Center, of which 2458 (87.6%) were enrolled in the hospital-based AGE surveillance. In the current study, we had access to 2240/2458 (91.1%) of these children, hospitalized between September 1, 2008, and October 31, 2015. Medical records of 187 children hospitalized between November 2007 and August 2008 were missing because of a cyber-attack, and records of 31 children were not found because of incorrect identification numbers.

The mean age of the participants was 16.3 months (SD = 13.9). Among the participants, 53.2% were males, and 52.6% were Arab children. Overall, 58.4% of the participants had a fever (≥38.0°C) at admission, 80.4% had vomiting, and 15.6% had dysentery. Rotavirus vaccine was given to 79.0% of children eligible for universal rotavirus vaccination (birth cohorts 2011–2015) (Table 1).

**Table 1.** Demographic and clinical characteristics of study participants with AGE.

	Included sample		Original sample	
**Characteristics**	**N**	**%**	**N**	**%**
**Overall**	2240	100%	2458	100%
**Age (months)**				
0-11	1042	46.5%	1141	46.4%
12-23	661	29.5%	729	29.7%
24-59	537	24.0%	588	23.9%
**Sex**				
Female	1049	46.8%	1145	46.6%
Male	1191	53.2%	1313	53.4%
**Ethnicity**				
Jews	1062	47.4%	1179	48.0%
Arab	1178	52.6%	1279	52.0%
**Residential socioeconomic status rank**				
1-3 (Low)	975	43.5%	1059	41.3%
4-5 (Intermediate)	808	36.1%	904	36.8%
6-10 (High)	303	13.5%	327	13.3%
Missing data	154	6.9%	168	6.8%
**Year of admission**				
November-December 2007	NA*	NA*	48	2.0%
2008**	91	4.1%	241	9.8%
2009	233	10.4%	237	9.6%
2010	431	19.2%	432	17.6%
2011	201	9.0%	203	8.3%
2012	421	18.8%	423	17.2%
2013	357	15.9%	358	14.6%
2014	340	15.2%	348	14.2%
2015	166	7.4%	168	6.8%
**Fever at admission (≥ 38°C)**	1308	58.4%	1415	57.6%
**Bloody stool (dysentery)**	350	15.6%	337	13.7%
**Vomiting**	1802	80.4%	1930	78.5%
**Rotavirus vaccination**				
Birth cohorts not eligible for the universal rotavirus vaccination 2007–2010	106/1085	9.8%	110/1280	8.6%
Birth cohorts eligible for universal rotavirus vaccination 2011–2015^§^	829/1050	79.0%	838/1062	78.9%

*NA=Not available. **Partial data were available for 2008, from 1 September to 31 December.

^§^Information on rotavirus vaccination was available for 2135 children. AGE= Acute gastroenteritis.

Stool culture was performed for 1911 (85.3%) participants; 370 (19.4%) had positive stool culture for *Campylobacter, Shigella*, or *Salmonella*. Rotavirus testing was performed in 1419 (63.3%) participants; 355 (25.0%) tested positive for rotavirus; 37.0% before the introduction of universal rotavirus vaccination (2008–2010), and 17.3% after the introduction of universal rotavirus vaccination (2011–2015).

Blood culture was performed among 877 (39.2%) of the participants, among whom only 1.3% had a positive culture result. Urine culture was performed on 242 (10.8%) participants; of those, 26.5% tested positive.

Chest X-ray imaging was performed for 360 (16.1%) children, of whom 19.4% had indications for bacterial pneumonia and 11.4% for viral pneumonia (Table 2).

**Table 2.** Microbiological cultures and chest X-ray imaging test of children aged 0–59 months hospitalized for acute gastroenteritis, 2008–2015.

Test	N	%
Overall	2240	100.0%
**Stool culture** performed	1911	85.3%
**Stool culture result (*n*=1911)**		
*Campylobacter*	249	13.0%
*Salmonella*	61	3.2%
*Shigella*	67	3.5%
Mixed infections*	7	0.04%
Any positive stool cultures	370	19.4%
Negative	1504	78.7%
Unknown	37	1.9%
**Rotavirus test** performed	1419	63.3%
**Rotavirus test result during the pre-universal rotavirus vaccination period 2008–2010 (*n*=557)**, Positive	206	37.0%
**Rotavirus test result during the universal rotavirus vaccination period 2011–2015 (*n*=862)**, Positive	149	17.3%
**Norovirus test** performed	653	29.2%
**Norovirus test results (*n*=653), positive**	79/653	12.1%
**Blood culture** performed	877	39.2%
**Blood culture result (*n*=877)**		
Positive**	11	1.3%
Negative	776	88.4%
Contamination	17	1.9%
Unknown	73	8.3%
**Urine culture** performed	242	10.8%
**Urine culture result (*n*=242)**		
*E. coli*	46	19.0%
*Klebsiella* (all species)^*¥*^	6	2.5%
Other^§^	12	5.0%
Unknown	26	10.7%
Negative	152	62.8%
**Chest x-ray imaging test** performed	360	16.1%
**Chest x-ray test results**		
Normal chest x-ray	232	64.4%
Chest x-ray of bacterial pneumonia	70	19.4%
Chest x-ray of viral pneumonia	41	11.4%
Other	1	0.3%
Unknown	16	4.5%

*Mixed infections in stool culture, *n*=5 *Campylobacter* and *Salmonella*, *n*=2 *Campylobacter* and *Shigella*.

**Positive blood culture: *Klebsiella n*=4, *Streptococcus spp*.n=3, *Kingella kingae n*=2, *Staphylococcus aureus n*=1, *E. Coli n*=1. ^¥^*Klebsiella* (all species) in urine culture: *Klebsiella pneumoniae n=*4, *Klebsiella oxytoca n*=2^.§^ Other urine culture results: *Proteus mirabilis n*=3, *Enterococcus n*=3; *Citrobacter koseri n*=1, *Pseudomonas aeruginosa n*=1, *Staphylococcus lugdunensis n*=1, *E. coli* and *Klebsiella n*=2.

The mean leukocyte level in blood count was 13.5 K/µl (SD = 6.1), and the mean neutrophils level was 7.6 K/µl (SD = 5.4). The median of CRP was 11.9 mg/L (IQR = 37.9) (Supplementary Table S2). Low sodium levels in the serum (<135 mEq/l) were found in 349/2128 (16.4%) children, and 58/1946 (3.0%) had low potassium levels (<3.5 mEq/l).

**Figure 1.**
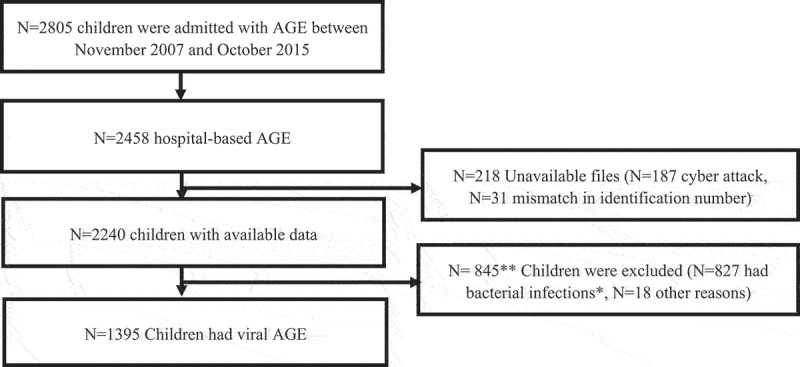
Flow chart.

Overall, 827 children with bacterial infections were eligible for receiving antibiotic treatment; 550 had bacterial AGE (a positive stool culture for *Shigella*, *Salmonella*, *Campylobacter*, or dysentery); 219 had bacterial co-infections with AGE, and 58 were classified as likely having a bacterial infection (Supplementary Table S3). These children were excluded from further analysis. The remaining 1395 children were classified as having viral AGE and were included in the current study, of those, 283 had RVGE, and 56 had norovirus AGE (Figure 1).

*Culture-proven bacterial gastroenteritis: positive stool culture for *Salmonella*, *Shigella*, or *Campylobacter*. Likely bacterial infection: blood leukocyte count> 15 K/µl, neutrophils level >10 K/µl, and C-reactive protein >50 mg/L. Bacterial co-infection: such as pneumonia, urinary tract infection, otitis media, etc. **Of 845 children with bacterial infections and other conditions who were excluded, 23 children tested positive for norovirus, and 72 children tested positive for rotavirus.

Among 1395 children with viral AGE, 86.8% had vomiting, and 36.8% had fever at admission, in addition to diarrhea. The median length of hospital stay was three days (IQR = 2). Chest X-ray imaging was performed in 146 (10.5%) of children with viral AGE; which was either interpreted as normal or consistent with a viral infection. Blood culture and urine culture were performed in 464 (33.2%) and 84 (6.0%), respectively, and all these cultures were negative.

### Antibiotic treatment among children hospitalized with viral AGE (2008–2015)

Antibiotic treatment was given to 253 of 1395 children with viral AGE, yielding an overall unnecessary antibiotic use of 18.1% (95% CI 16.2–20.2). Antibiotic treatment before admission was given to 103/253 children (40.7% [95% CI 34.8–46.9]) who were treated with antibiotics. Overall, 174 of 253 children received antibiotics during hospitalization (68.8% [95% CI 62.9–74.3]), and 67/253 (38.5% [95% CI 21.3–32.2]) had prescriptions on discharge for continuing treatment. The most common antibiotic treatment was penicillin given to 118/253 (46.6%), followed by the third-generation cephalosporin ceftriaxone 86/253 (34.0%), and macrolides, namely azithromycin 55/253 (21.7%). Other antibiotic agents were also given, although less frequently, including amoxicillin/clavulanic acid, cefuroxime, and metronidazole, to 9.1%, 8.3%, and 4.3% of the treated children (Table 3).

**Table 3.** Antibiotic agents prescribed for children hospitalized with viral AGE (*n* = 1395) overall and by timing relative to hospital admission (2008–2015).

	Antibiotics treatment (any)	Before admission	During hospitalization	At discharge
	N (%)	N (%)	N (%)	N (%)
**Received antibiotics**				
**Yes**	253/1395 (18.1%)	103/253 (40.7%)	174/253 (68.8%)	67/253 (38.5%)
**No**	1142/1395 (81.9%)	NA	NA	NA
**Antibiotic agents**^**§**^				
**Penicillin**				
Ampicillin	6/253 (2.4%)	0/103 (0.0%)	6/174 (3.4%)	0/67 (0.0%)
Amoxicillin	85/253 (33.6%)	49/103 (47.6%)	18/174 (10.3%)	38/67 (56.7%)
Penicillin V	2/253 (0.8%)	0/103 (0.0%)	1/174 (0.6%)	2/67 (3.0%)
Penicillin	1/253 (0.4%)	0/103 (0.0%)	1/174 (0.6%)	0/67 (0.0%)
Amoxicillin/clavulanic acid	23/253 (9.1%)	14/103 (13.6%)	3/174 (1.7%)	6/67 (9.0%)
Piperacillin/tazobactam	1/253 (0.4%)	0/103 (0.0%)	1/174 (0.6%)	0/67 (0.0%)
**Cephalosporins 1st generation**				
Cefamezin	2/253 (0.8%)	0/103 (0.0%)	3/174 (1.7%)	0/67 (0.0%)
Cephalexin	13/253 (5.1%)	6/103 (5.8%)	4/174 (2.3%)	6/67 (9.0%)
**Cephalosporins 2nd generation**				
Cefuroxime	21/253 (8.3%)	3/103 (2.9%)	17/174 (9.8%)	3/67 (4.5%)
**Cephalosporins 3rd generation**				
Ceftriaxone	86/253 (34.0%)	1/103 (1.0%)	85/174 (48.9%)	0/67 (0.0%)
**Macrolides**				
Azithromycin	55/253 (21.7%)	34/103 (33.0%)	21/174 (12.1%)	11/67 (16.4%)
**Other antibiotics**				
Gentamicin	9/253 (3.6%)	0/103 (0.0%)	9/174 (5.2%)	0/67 (0.0%)
Metronidazole	11/253 (4.3%)	1/103 (1.0%)	11/174 (6.3)	2/67 (3.0%)
Clindamycin	4/253 (1.6%)	0/103 (0.0%)	4/174 (2.3%)	0/67 (0.0%)
Vancomycin	1/253 (0.4%)	0/103 (0.0%)	1/174 (0.6%)	0/67 (0.0%)
**Unknown**	24/253 (9.5%)	6/103 (5.8%)	18/174 (10.3%)	0/67 (0.0%)

^**§**^Some received more than one antibiotic agent; therefore, the percentages exceed 100%. The percentages were calculated among children who received antibiotics.

AGE: Acute gastroenteritis. NA: not applicable.

### Correlates of unnecessary antibiotic treatment among children with viral AGE

Antibiotics were prescribed during hospitalization and on discharge for 182 (13.0% [95% CI 11.4–14.9]) children with viral AGE. There were significant differences in the distribution of the years of admission between children with unnecessary antibiotic treatment (*p* < .001) compared to children who did not receive antibiotics. The proportions of children hospitalized from 2008 to 2011 were lower and ranged between 1.1% to 13.2% in the treated group compared to 4.7% to 23.3% in the untreated group, while the proportions of children hospitalized during 2012, 2014, and 2015 were higher in the treated (26.4%, 20.9%, and 10.4%, respectively) versus the untreated group (17.6%, 11.9%, and 6.3%, respectively). The treated and untreated groups were similar regarding the proportion of hospitalizations that occurred during 2013: 14.8% and 16.2%, respectively. Fever was more common in children with unnecessary antibiotic treatment versus the untreated children (49.5% versus 35.2%, *p* < .001), whereas vomiting was less common in the former group (80.2% versus 87.8%, *p* = .005). Universal rotavirus vaccination was introduced in Israel in December 2010. Therefore, the analysis of the association between rotavirus vaccination and unnecessary antibiotic use was limited for those who were eligible for universal rotavirus vaccination i.e., birth cohort between 2011 and 2015. The proportion of children who were vaccinated for rotavirus was lower among children who were treated with antibiotics versus those who were not: 71.2% versus 82.0% (*p* = .01). The proportion of children in whom chest X-rays were performed was significantly higher in children who received antibiotics, 33.0% versus 7.1% in children who did not (*p* < .001). Similarly, the proportions of children in whom blood cultures (56.0% versus 29.8%) and urine culture (24.2% versus 3.3%) were performed were higher in the former group (*p* < .001). Testing positive for rotavirus was significantly lower in children treated with antibiotics, 17.3% versus 34.4% in children who were not (*p* < .001).

Compared to children who were not treated with antibiotics, children with unnecessary antibiotic treatment had higher mean levels of platelets (375.6 K/µl and 403.6 K/µl, respectively, p < .001), and leukocytes (12.4 K/µl versus 14.4 K/µl *p *< .001), and higher median CRP level (6.4 mg/L versus 17.6 mg/L (*p* < .001). No significant differences were found between the groups in age (*p *= .077), sex (*p* = .731), ethnicity (p = .505), SES (*p *= .463), previous AGE hospitalization (*p* = .071), rotavirus test (*p *= .467), levels of hemoglobin (*p* = .701), neutrophils (*p* = .057), lymphocytes (*p* = .255), glucose (*p* = .288), BUN (*p *= .5), and creatinine (*p* = .483) (Table 4).

**Table 4.** Demographic and clinical factors associated with unnecessary antibiotic use in children hospitalized with viral AGE- Bivariate analysis.

	Antibiotic treatment during hospitalization N=182	No antibiotic treatment during hospitalization *N*=1213	P value
Age (months), N (%)			.077
0-11	90 (49.5%)	526 (43.4%)	
12-23	44 (24.2%)	394 (32.5%)	
24-59	48 (26.4%)	293 (24.2%)	
Sex, males, N (%)	97 (53.3%)	663 (54.7%)	.731
Ethnicity (Jewish vs. Arabs), N (%)	87 (47.8%)	612 (50.5%)	.505
Residential socioeconomic status rank, N (%)			.463
1-3 (Low)	77 (44.0%)	494 (44.0%)	
4-5 (Intermediate)	75 (42.9%)	443 (39.4%)	
6-10 (High)	23 (13.1%)	186 (16.6%)	
Year of admission, N (%)			<.001
Sep-Dec-2008	2 (1.1%)	57 (4.7%)	
2009	11 (6.0%)	129 (10.6%)	
2010	24 (13.2%)	283 (23.3%)	
2011	13 (7.1%)	114 (9.4%)	
2012	48 (26.4%)	214 (17.6%)	
2013	27 (14.8%)	196 (16.2%)	
2014	38 (20.9%)	144 (11.9%)	
Jan-Oct-2015	19 (10.4%)	76 (6.3%)	
Background diseases, N (%)	39 (21.4%)	201 (16.6%)	.012
Previous lifetime hospitalization, N (%)	95 (52.2%)	483 (39.8%)	.002
Previous AGE hospitalization, N (%)	38 (20.9%)	189 (15.6%)	.071
Fever on admission, N (%)	90 (49.5%)	424 (35.2%)	<.001
Vomiting, N (%)	146 (80.2%)	1065 (87.8%)	.005
Number of stools on the most severe day, N (%)			
0-5	99 (54.4%)	559 (46.1%)	.036
≥6	83 (45.6%)	654 (53.9%)	
Receiving rotavirus vaccine*, N (%)	79 (71.2%)	409 (82.0%)	.01
Chest-X-ray test performed, N (%)	60 (33.0%)	86 (7.1%)	<.001
Blood culture performed, N (%)	102 (56.0%)	362 (29.8%)	<.001
Urine culture performed, N (%)	44 (24.2%)	40 (3.3%)	<.001
Rotavirus test performed, N (%)	110 (60.4%)	767 (63.2%)	.467
Rotavirus test results^§^, N (%)			<.001
Positive	19 (17.3%)	264 (34.4%)	
Negative	87 (79.1%)	438 (57.1%)	
Unknown	4 (3.6%)	65 (8.5%)	
Hemoglobin (g/dl), mean (SD)^#^	11.6 (1.3), *N*=179	11.8 (1.4), *N*=1141	.701
Platelets (K/µl), mean (SD)^#^	403.6 (169.8), *N*=179	375.6 (128.9), *N*=1142	<.001
Leukocytes (K/µl), mean (SD)^#^	14.4 (7.3), *N*=179	12.4 (5.2), *N*=1143	<.001
Neutrophils (K/µl), mean (SD)^#^	7.9 (5.4), *N*=177	6.9 (4.5), *N*=1140	.057
Lymphocytes(K/µl), mean (SD)^#^	4.6 (2.5), *N*=178	4.3 (2.9), *N*=1140	.255
Glucose (mg/dL), mean (SD)^#^	94.7 (25.9), *N*=170	86.7 (22.6), *N*=1131	.288
Blood urea nitrogen(mg/dL), mean (SD)^#^	11.4 (6.8), *N*=174	12.9 (5.9), *N*=1141	.5
Creatinine(mg/dL), mean (SD)^#^	0.3 (0.1), *N*=172	0.3 (0.2), *N*=1108	.483
C-reactive protein (mg/L), median (IQR)^#^	17.6 (51.6), *N*=149	6.4 (15.3), *N*=1060	<.001
Sodium mEq/l, mean (SD)^#^	138.2 (4.8), *N*=174	138.3 (3.7), *N*=1140	.035
Potassium mEq/l, mean (SD)^#^	4.5 (0.8), *N*=155	4.6 (0.6), *N*=1045	.002

*This analysis is based on children with viral AGE eligible for rotavirus vaccination in the universal vaccination program – birth cohorts 2011–2015. ^§^This analysis included only children who performed rotavirus test. ^#^The numbers (N) represent participants with available data. AGE= Acute gastroenteritis; IQR= Interquartile range; SD=Standard deviation.

A multivariable logistic regression model showed that the likelihood of receiving unnecessary antibiotic treatment was higher in children who performed urine culture (adjusted OR = 7.12, 95% CI 3.77–13.43), blood culture (adjusted OR = 3.29, 95% CI 1.85–5.86) and chest X-ray imaging (adjusted OR = 3.00, 95% CI 1.73–5.23) compared to children who were not tested. The odds of receiving unnecessary antibiotic treatment increased by 2% with each 1 mg/L increase in CRP (adjusted OR = 1.02, 95% CI 1.01–1.02) and by 5% for every 1 K/µl increase in blood leukocyte count (adjusted OR = 1.05, 95% CI 1.01–1.09). Rotavirus vaccination was negatively associated with unnecessary antibiotic use (adjusted OR = 0.53, 95% CI 0.31–0.91). Age, residential SES rank, fever, vomiting, and number of stools were not significantly associated with unnecessary antibiotic use in this analysis (Model 1 Table 5). The values of VIF were mostly around 1.0, suggesting no multicollinearity between the independent variables.

**Table 5.** Logistic regression models of factors associated with unnecessary antibiotic use in children with viral AGE (2008–2015).

			Model 1^a^		Model 2^b^	
Variable	Unadjusted OR (95% CI)	P value	Adjusted OR (95% CI)	P value	Adjusted OR (95% CI)	P value
Age (months), continuous variable	1.00 (0.98–1.01)	.429	0.99 (0.98–1.01)	.444	0.99 (0.97–1.00)	.129
Residential socioeconomic status rank	Df=2	.465	Df=2	.177	Df=2	.040
1-3 (Low)	Reference		Reference		Reference	
4-5 (Intermediate)	1.09 (0.77–1.53)	.637	1.33 (0.84–2.09)	.221	1.41 (0.84–2.37)	.191
6-10 (High)	0.79 (0.48–1.30)	.360	0.74 (0.38–1.45)	.376	0.49 (0.21–1.15)	.100
Fever at admission, yes vs. no	1.80 (1.31–2.46)	<.001	1.03 (0.66–1.61)	.897	0.82 (0.49–1.39)	.467
Vomiting, yes vs. no	0.56 (0.38–0.84)	.005	1.57 (0.84–2.93)	.161	1.43 (0.71–2.88)	.324
Leukocytes (K/µl), continuous variable	1.06 (1.03–1.08)	<.001	1.05 (1.01–1.09)	.018	1.05 (1.01–1.10)	.017
C-reactive protein (mg/L), continuous variable	1.02 (1.02–1.03)	<.001	1.02 (1.01–1.02)	<.001	1.02 (1.00–1.04)	.056
Rotavirus vaccination (yes vs. no)	0.54 (0.34–0.87)*	.011	0.53 (0.31–0.91)	.022	0.41 (0.22–0.75)	.004
**Year of admission**	Df=7	<.001	Df=7	.002	Df=7	<.001
2008	0.14 (0.03–0.63)	.010	1.37 (0.21–9.02)	.743	3.12 (0.23–42.44)	.393
2009	0.34 (0.15–0.76)	.008	1.45 (0.25–8.65)	.681	3.30 (0.27–40.40)	.350
2010	0.34 (0.17–0.65)	.001	1.99 (0.32–12.53)	.463	4.12 (0.32–53.35)	.279
2011	0.46 (0.21–0.98)	.044	4.55 (0.78–26.55)	.093	11.30 (0.94–135.41)	.056
2012	0.90 (0.50–1.62)	.720	0.92 (0.15–5.79)	.929	1.75 (0.14–22.07)	.667
2013	0.55 (0.29–1.05)	.070	1.95 (0.32–12.08)	.471	2.79 (0.23–34.46)	.423
2014	1.06 (0.57–1.96)	.864	1.95 (0.28–13.42)	.499	3.69 (0.27–51.53)	.331
2015	Reference		Reference		Reference	
Chest-x-ray test performed, yes vs. no	6.45 (4.41–9.41)	<.001	3.00 (1.73–5.23)	<.001	4.72 (2.44–9.14)	<.001
Blood culture performed, yes vs. no	3.00 (2.18–4.12)	<.001	3.29 (1.85–5.86)	<.001	3.79 (1.99–7.21)	<.001
Urine culture performed, yes vs. no	9.35 (5.88–14.86)	<.001	7.12 (3.77–13.43)	<.001	8.27 (4.09–16.69)	<.001
Number of stools ≥6 vs. 0–5	0.72 (0.52–0.98)	.037	0.89 (0.57–1.38)	.592	0.87 (0.52–1.45)	.596

^a^Model 1 included 1047 children with viral AGE and available data on the study variables that were included in the analysis as shown in the table, Nagelkerke R^2^=0.290. ^b^ Model 2 included children with viral AGE who had CRP levels equal to or less than 50 mg/L, *N*=944, with available data on the study variables that were included in the analysis, as shown in the table. *This analysis included children eligible for receiving rotavirus Nagelkerke R^2^=0.287. AGE= Acute gastroenteritis; CI=Confidence interval; Df=Degree of freedom; OR=Odds ratio.

### Sensitivity analyses

Excluding from the analysis 119 children with CRP levels >50 mg/L (but their leukocyte level ≤ 15 K/µl and neutrophil level ≤ 10 K/µl) showed that 141/1276 (11.1% [95% CI 9.4–12.9]) with viral AGE received antibiotics. Repeating the bivariate analysis in children with viral AGE and CRP ≤50 mg/L yielded similar results to those in the primary analysis of all children with viral AGE. In addition, this analysis showed that a previous hospitalization and having background diseases were significantly more common in children with unnecessary antibiotic use compared to untreated children (52.5% versus 39.7%, *p* = .004) and 19.9% versus 16.5%, (*p* = .010), and the mean levels of glucose and potassium were higher in the treated group (*p* = .005) (Supplementary Table S4). The results of the logistic regression analysis (model 2 Table 5) were similar to those found in the main analysis, except that CRP level was not significantly related to unnecessary antibiotic use.

Limiting the analysis to birth cohorts who were eligible for receiving rotavirus vaccination during the universal vaccination years showed that 114/621 (18.4% [95% CI 15.5–21.6%) received antibiotics. The bivariate analysis based on this group yielded consistent results to those found in analyses of all children with viral AGE and those with CRP levels ≤50 mg/L (Table 6).

**Table 6.** Factors associated with unnecessary antibiotic use in children hospitalized with viral AGE who belonged to birth cohorts eligible for rotavirus vaccination during the universal vaccination years 2011–2015 - bivariate analysis.

	Antibiotic treatment during hospitalization N=114	No antibiotic treatment during hospitalization *N*= 507	P value
Age (months), N (%)			.718
0-11			
12-23	70 (61.4%)	302 (59.6%)	
24-59	33 (28.9%)	164 (32.3%)	
Sex, males, N (%)	11 (9.6%)	41 (8.1%)	.350
Ethnicity (Jewish vs. Arabs), N (%)	57 (50.0%)	278 (54.8%)	.234
Residential socioeconomic status rank, N (%)	53 (46.5%)	267 (52.7%)	.548
1-3 (Low)			
4-5 (Intermediate)	49 (45.4%)	198 (42.1%)	
6-10 (High)	47 (43.5%)	201 (42.8%)	
Year of admission, N (%)	12 (11.1%)	71 (15.1%)	.223
2011			
2012	4 (3.5%)	28 (5.5%)	
2013	35 (30.7%)	129 (25.4%)	
2014	22 (19.3%)	146 (28.8%)	
2015	34 (29.8%)	130 (25.6%)	
Background diseases, N (%)	19 (16.7%)	74 (14.6%)	.190
Previous lifetime hospitalization, N (%)	23 (20.2%)	77 (15.2%)	.227
Previous AGE hospitalization, N (%)	62 (54.4%)	244 (48.1%)	.958
Fever on admission, N (%)	20 (17.5%)	90 (17.8%)	.004
Vomiting, N (%)	60 (52.6%)	192 (37.9%)	.104
Number of stools on the most severe day, N (%)	87 (76.3%)	420 (82.8%)	.062
0-5			
≥6	71 (62.3%)	267 (52.7%)	
Received rotavirus vaccine, N (%)	43 (37.7%)	240 (47.3%)	.010
Chest-X-ray test performed, N (%)	79 (71.2%)	409 (82.0%)	<.001
Blood culture performed, N (%)	43 (37.7%)	35 (6.9%)	.002
Urine culture performed, N (%)	80 (70.2%)	275 (54.2%)	<.001
Rotavirus test performed, N (%)	34 (29.8%)	26 (5.1%)	.973
Rotavirus test results^§^, N (%)	69 (60.5%)	306 (60.4%)	.067
Positive			
Negative	6 (8.7%)	56 (18.3%)	
Unknown	63 (91.3%)	244 (79.7%)	
Hemoglobin (g/dl), mean (SD)^#^	0 (0.0%)	6 (2.0%)	.631
Platelets (K/µl), mean (SD)^#^	11.4 (1.3), *N*=112	11.5 (1.3), *N*=484	.10
Leukocytes (K/µl), mean (SD)^#^	416.7 (175.0), *N*=122	378.9 (129.3), *N*=484	<.001
Neutrophils (K/µl), mean (SD)^#^	14.8 (7.8), *N*=112	12.3 (4.9), *N*=484	.001
Lymphocytes(K/µl), mean (SD)^#^	7.5 (5.6), *N*=110	6.0 (4.0), *N*=482	.455
Glucose (mg/dL), mean (SD)^#^	5.1 (2.6), *N*=111	4.9 (2.6), *N*=482	.003
Blood urea nitrogen(mg/dL), mean (SD)^#^	95.3 (20.5), *N*=104	88.8 (19.5), *N*=481	.141
Creatinine(mg/dL), mean (SD)^#^	11.0 (7.2), *N*=107	11.9 (5.7), *N*=484	.078
C-reactive protein (mg/L), median (IQR)^#^	0.3 (0.1), *N*=106	0.3 (0.1), *N*=471	<.001
Sodium mEq/l, mean (SD)^#^	18.6 (51.0), *N*=91	6.6 (15.4), *N*=444	.675
Potassium mEq/l, mean (SD)^#^	138.4 (5.2), *N*=107	138.3 (3.5), *N*=484	.140
	4.7 (0.7), *N*=99	4.6 (0.6), *N*=459	

^§^This analysis included only children who performed rotavirus test. ^#^ The numbers (N) represent participants with available data. AGE= Acute gastroenteritis; IQR= Interquartile range; SD=Standard deviation.

A multivariable model based on these birth cohorts also showed positive associations between CRP, blood leukocyte levels, year of admission, performing chest X-ray test, blood culture, and urine culture with unnecessary antibiotic use, while rotavirus vaccination was inversely associated with unnecessary antibiotic treatment (Table 7). The results were similar when limiting the analysis to the 2012–2015 birth cohorts, i.e., considering 2011 as a transitional year (Supplementary Tables S5 and S6).

**Table 7.** Logistic regression analysis of factors associated with unnecessary antibiotic use in children with viral AGE who belong to birth cohorts (2011–2015) eligible for receiving rotavirus vaccination during the universal vaccination years.

Variable	Unadjusted OR (95% CI)	P value	Adjusted OR (95% CI)	P value
Leukocytes (K/µl), continuous variable	1.07 (1.04–1.11)	<.001	1.06 (1.01–1.11)	.029
C-reactive protein (mg/L), continuous variable	1.03 (1.02–1.04)	<.001	1.02 (1.02–1.04)	<.001
Rotavirus vaccination (yes, vs. no)	0.54 (0.34–0.87)	.011	0.42 (0.21–0.87)	.019
**Year of admission**	Df=4	.232	Df=4	.005
2011	Reference		Reference	
2012	1.90 (0.63–5.78)	.258	3.82 (0.83–17.63)	.086
2013	1.06 (0.34–3.30)	.927	0.74 (0.15–3.77)	.720
2014	1.83 (0.60–5.58)	.287	1.14 (0.23–5.55)	.872
2015	1.80 (0.56–5.75)	.323	1.37 (0.26–7.28)	.964
Chest-X-ray test performed, yes vs. no	8.17 (4.90–13.62)	<.001	3.98 (2.00–7.90)	<.001
Blood culture performed, yes vs. no	1.99 (1.28–3.08)	.002	2.11 (1.03–4.34)	.042
Urine culture performed, yes vs. no	7.86 (4.48–13.80)	<.001	6.88 (3.26–14.54)	<.001

*N* = 519, Nagelkerke R2 = 0.371. AGE = Acute gastroenteritis; CI = Confidence interval; Df = Degree of freedom; OR = Odds ratio.

Limiting the analysis to children with RVGE or norovirus AGE showed that unnecessary antibiotic treatment was given to 25/334 (7.4% [95% CI 5.0–10.7]) children. The bivariate analysis yielded results consistent with those found in prior analyses. In addition, residential SES rank was positively associated with unnecessary antibiotic use (*p* = .042), background diseases were more common in children treated with unnecessary antibiotics compared to the untreated ones (28.0% versus 16.2%, *p* < .001), as well as a history of a previous AGE hospitalization (28.0% versus 12.9%, *p* = .037). Ethnicity was borderline significantly associated with antibiotic use (*p* = .059). The mean potassium level in children who received antibiotics was 4.8 mEq/l versus 4.5 mEq/l in children who did not receive antibiotics (*p* = .048) (Supplementary Table S7).

A multivariable logistic regression showed a lower likelihood of receiving unnecessary antibiotics in relation to rotavirus vaccination (OR = 0.22 95% CI 0.06–0.76). This model also showed a positive association between performing a blood culture, urine culture or chest X-ray test and receiving unnecessary antibiotic treatment (*p* < .001). Jewish children exhibited a lower likelihood of receiving unnecessary antibiotics compared to Arab children (adjusted OR = 0.28, 95% CI 0.09–0.86). The associations of previous AGE hospitalization and CRP with unnecessary antibiotics use were not significant (Table 8).

**Table 8.** Logistic regression analysis of factors associated with unnecessary antibiotic use in children hospitalized with laboratory-confirmed RVGE or norovirus AGE.

Variable	Unadjusted OR (95% CI)	P-value	Adjusted OR (95% CI)	P-value
Ethnicity (Jewish vs. Arab)	0.43 (0.17–1.06)	.065	0.28 (0.09–0.86)	.026
Rotavirus vaccination (yes vs. no)	0.50 (0.11–2.15)*	.348	0.22 (0.06–0.76)	.017
Previous AGE hospitalization yes vs. no	2.11 (0.93–4.78)	.075	3.13 (0.99–9.95)	.053
C-reactive protein (mg/L), continuous variable	1.02 (1.00–1.04)	.020	1.02 (1.0–1.04)	.093
Performed blood or urine culture or chest X-ray test, yes vs. no	7.39 (3.06–17.85)	<.001	9.42 (3.23–27.51)	<.001

N = 283, Nagelkerke R^2^ = 0.267. *This analysis included children eligible for receiving rotavirus vaccine. AGE = Acute gastroenteritis; CI = Confidence interval; OR = Odds ratio; RVGE = Rotavirus gastroenteritis.

Performing chest X-ray imaging and blood or urine culture might suggest a more severe illness; therefore, we compared clinical characteristics between children with viral AGE in whom these tests were performed and those who were not. Children with chest X-ray test had fever on admission significantly more often than children in whom this test was performed: 69.7% vs. 34.4%, but less often suffered from vomiting (73.3% vs. 88.4%) or had ≥ 6 stools on the most severe day (39.3% vs. 54.8%), *p* < .001 for all comparisons. Children in whom a chest X-ray imaging was performed also had urine culture (15.8% vs. 4.9%, *p* < .001) and blood culture (52.1% vs. 31.1%, *p* < .001) performed more often but were less likely to be tested for rotavirus (52.4% vs. 64.0%) or have a positive rotavirus test result (16.7% vs. 33.8%). Similarly, children who had a blood culture had fever on admission more often than children in whom a blood culture was not performed (52.4% vs. 29.4%), had less often vomiting (81.5% vs. 89.5%), and ≥ 6 stools in the most severe day (43.5% vs. 58.0%), *p* < .001 for all comparisons. Moreover, children in whom a blood culture was performed had more often chest X-ray imaging and urine culture (*p* < .001 for both comparisons). However, they were less frequently tested for rotavirus (*p* = .005). Similar findings were observed when comparing children in whom urine culture was performed urine culture to those who were not (Supplementary Tables S8, S9 and S10). Sensitivity analyses in which we excluded children in whom chest X-ray imaging and urine culture were performed, showed that antibiotic treatment was given to 153/1188 (12.9%) of these children. The results of the multivariable model were consistent with the primary analysis, showing significant positive associations between leukocytes and CRP levels and a strong inverse association with receipt of the rotavirus vaccine (supplementary tables S11 and S12). A further exclusion of children in whom a blood culture was performed, showed that 88/841 (10.9%) of these patients had unnecessary antibiotic treatment. CRP level was positively related to unnecessary antibiotic treatment, while a strong inverse association was found with receipt of the rotavirus vaccine in this group as well (Supplementary tables S13 and S14).

## Discussion

In this study, we characterized unnecessary antibiotic treatment in children hospitalized for viral AGE and examined the correlates of unnecessary antibiotic use, including the role of rotavirus vaccination. We found that 18.1% of children with viral AGE received unnecessary antibiotic treatment, which was similar (18.4%) in birth cohorts of the universal rotavirus vaccination years. Excluding children with CRP >50 mg/L (possible bacterial infections) or limiting the analysis to those who tested positive for rotavirus or norovirus showed lower antibiotic prescriptions of 11.1% and 7.4% of children, respectively, which highlights the role of laboratory diagnostics in reducing antibiotic use. In a previous study focusing on bacterial AGE, we have shown that 67.1% of children hospitalized with bacterial AGE received appropriate antibiotic treatment, mainly ceftriaxone and azithromycin.^41^ Studies from low-middle-income countries showed that a high proportion of antibiotic-treated diarrheal episodes was attributed to rotavirus; 8.6% in the Etiology, Risk Factors and Interactions of Enteric Infections and Malnutrition and the Consequences for Child Health and Development Study (MAL-ED)^11^ and 29.2% in the GEMS.^5^ While most prior studies of antibiotic use in children with AGE were undertaken in low-middle-income countries, our study is novel by focusing on children from a high-income country setting with relatively strict prescription patterns and antibiotic stewardship programs.

In the current study, the most common antibiotic treatment given to children hospitalized for viral AGE was penicillin (46.6%), followed by ceftriaxone (34.0%) and azithromycin (21.7%), other antibiotics were also given including amoxicillin/clavulanic acid (9.1%), cefuroxime (8.3%) and metronidazole (4.3%). While ceftriaxone and azithromycin are indicated for bacterial AGE treatment,^65–69^ amoxicillin is usually prescribed for the treatment of respiratory infections.^70,71^ This suggests that children were empirically treated with ceftriaxone and azithromycin due to suspected bacterial AGE, while treatment with other antibiotics was likely medically unjustified. A study from Nigeria involving children aged less than five years with acute watery diarrhea^13^ showed that more than 90% of the participants had rotavirus diarrhea.^13^ In that study, antibiotics were administrated to 86.9% of children, mostly ciprofloxacin (72.4%), metronidazole (30.2%), and gentamicin (15.1%).^13^ The MAL-ED study showed that fluoroquinolone (33.0%) and macrolides (28.0%) were the most prescribed antibiotics for diarrhea.^11^

In the GEMS, trimethoprim/sulfamethoxazole was the most common antibiotic prescribed in the African sites, whereas quinolones were more prevalent in the South Asian sites, and azithromycin was common in Bangladesh.^5^

Our study demonstrated a significant decline in the percentage of children with positive rotavirus tests after the introduction of universal rotavirus vaccination, from 37.0% before the universal vaccination years (2008–2010) to 17.3% in the universal vaccination years (2011–2015), as we and others have shown.^28,36,38,48,51^ We also found that rotavirus vaccination was inversely associated with unnecessary antibiotics use in children with viral AGE with OR of 0.41 to 0.54 in the various analyses, these findings were independent of indicators of disease severity such as CRP and leukocyte levels, performing blood and urine culture and chest X-ray. This novel finding is based on real-world data and corroborates a previous study from the United States.^39^ While the study from the United States relied on data from a commercial database,^39^ we have comprehensively reviewed individual medical records of active surveillance of RVGE. The finding of an inverse association between rotavirus vaccination and unnecessary antibiotic use in children with AGE found in our study and reported by Hall et al. in the United States is in agreement with a modeling study,^15^ suggesting that universal rotavirus vaccination programs might reduce the likelihood of unnecessary antibiotic consumption in patients with AGE in low-middle and high income countries. Collectively our and others’ findings^15,39^ highlight additional benefits of rotavirus vaccination of reducing antibiotic use. Rotavirus vaccines were shown to be effective in preventing RVGE, including severe disease.^28,34,38,47,48,72^ Therefore, there is a possibility that the rotavirus vaccine reduces the severity of illness, which might affect the physician’s clinical judgment regarding the need for antibiotic treatment. Previous studies on influenza vaccines have also shown that the administration of influenza vaccine was related to a decrease in unnecessary antibiotic consumption,^73–76^ which was also explained by a decline in influenza-related illnesses through vaccination. This vaccine impact might lead to a reduction in antibiotic prescriptions. Together, these findings imply that vaccines play a pivotal indirect role in combating antibiotic resistance by reducing antibiotic use. Thus, the indirect gains from vaccines might be greater than assumed.

We found positive associations between levels of CRP and blood leukocytes and unnecessary antibiotic treatment, corroborating findings that were reported in studies conducted in South Korea and Italy^77,78^ and our study on antibiotic treatment in children with bacterial AGE.^41,77^ Our study also demonstrated that performing blood and urine cultures, and a chest X-ray test, were positively associated with unnecessary antibiotic treatment. Prior studies on antibiotic use in children with AGE did not address such variables. Thus, our study sheds light on the clinical decision-making process in real life. Comparable results were reported for performing such tests in a retrospective study involving hospitalized children aged 0–24 months with respiratory syncytial virus^79^ and children hospitalized with bacterial AGE.^41^ Performing blood culture, urine culture, and chest X-ray indicates a physician’s perception of severe infection and clinical suspicion of bacterial infection. Therefore, sensitivity analyses excluded children in whom blood or urine culture or chest X-ray tests were performed. Indeed, in these analyses, the percentage of children with unnecessary antibiotic treatment declined to 10.9%, but the results were consistent, showing an inverse association between rotavirus vaccination and unnecessary antibiotic treatment, and a positive association with CRP level.

Following the implementation of the universal rotavirus vaccine, a declining trend in the odds of unnecessary antibiotic treatment for children with viral AGE was observed. However, it is surprising that antibiotic use was high in the year 2012 despite this trend.

The findings regarding the correlates of unnecessary antibiotics use in children with viral AGE were consistent across several sensitivity analyses, such as limiting the analysis to children with viral AGE who had CRP level ≤50 mg/L, birth cohorts eligible for receiving rotavirus vaccination during the universal vaccination era, and children with laboratory-confirmed rotavirus or norovirus AGE. In the latter, we also found an association between ethnicity and unnecessary antibiotic use, suggesting that Jewish children exhibited a lower likelihood of receiving unnecessary antibiotics compared to Arab children.

There are several reasons for unnecessary antibiotic use in children with viral AGE. There were a few children who started antibiotic therapy in the community before admission to the hospital and continued the treatment course during hospitalization. In other patients, unnecessary antibiotic use was most likely driven by clinical manifestation or partial laboratory test results indicating more severe illness, such as CRP and leukocyte levels, before culture results became available. Educational interventions, including recurrent updates regarding the guidelines for antibiotic treatment in children with AGE, are warranted to reduce unnecessary antibiotic use. Moreover, introducing rapid diagnostic testing, such as molecular diagnosis, can assist in clinical decision-making regarding the necessity of antibiotic treatment in children with AGE.

Our study has limitations. Data on some of the variables were obtained retrospectively from medical records, including on antibiotic use. Thus, there might be variability and incomplete documentation. To assure completeness we screened the entire medical record. Since antibiotic treatments can be given only with a physician’s prescription, it is unlikely that we missed children who were treated with antibiotics due to a lack of documentation. The study was conducted in a single medical center; thus, the generalizability of the rate of unnecessary antibiotic use in children with viral AGE might be limited. Physician decisions might be different among medical centers, but we assume that antibiotic prescription patterns adhere to standard guidelines. Moreover, the study center is similar to other governmental public hospitals in Israel. The findings regarding the correlates of unnecessary antibiotic use in children with viral AGE can be generalizable to other populations with comparable characteristics and healthcare systems, for example, in high-income countries. Stool samples in our study were not tested for the presence of Enterotoxigenic *Escherichia coli* (ETEC) since it is not included in the routine testing in patients with AGE; thus, there might be some misclassification of the viral AGE infections. However, this is not expected to affect the results regarding the correlates of unnecessary antibiotic use.

The strengths of our study include the long study period spanning over seven years, which allowed extensive data collection, and the large sample size. Furthermore, data obtained from different sources (e.g., various parts in the medical record, parental interviews, and laboratory tests) were used for cross-validation, thus enhancing the validity of the combined dataset. Information on antibiotic prescriptions is highly accurate since antibiotics in Israel can be prescribed only by physicians and should be documented in the medical records so that nurses can give antibiotic treatment according to the prescription. The study population represented well both the Jewish and Arab populations and various socioeconomic status levels, enhancing the generalizability of our findings. Moreover, laboratory tests, including complete blood count, blood and urine culture, rotavirus test, chest X-ray, and stool culture for over 80% of participants, were conducted according to standard methods and by qualified and experienced laboratory staff. The results of these tests supported the classification of viral and bacterial infections and reduced potential misclassification of unnecessary antibiotic use. Information on the rotavirus vaccine was obtained through linkage with the national immunization registry at the Ministry of Health, thereby enhancing the completeness and validity of data on rotavirus vaccination

## Conclusions

Unnecessary antibiotic prescriptions were common in children hospitalized for viral AGE. The correlates of unnecessary antibiotic prescriptions for viral AGE suggested clinical judgment of severe illness. The inverse association between rotavirus vaccination and antibiotic treatment might indicate less severe illness in vaccinated children, thus highlighting the indirect role of vaccines in reducing unnecessary antibiotic use, which contributes to the spread of antimicrobial resistance.

## Supplementary Material

Supplementary material final.docx

## References

[1] Kotloff KL, Nataro JP, Blackwelder WC, Nasrin D, Farag TH, Panchalingam S, Wu Y, Sow SO, Sur D, Breiman RF, et al. Burden and aetiology of diarrhoeal disease in infants and young children in developing countries (the global enteric multicenter study, GEMS): a prospective, case-control study. Lancet. 2013;382(9888):209–14. doi:10.1016/S0140-6736(13)60844-2. 23680352

[2] Ugboko HU, Nwinyi OC, Oranusi SU, Oyewale JO. Childhood diarrhoeal diseases in developing countries. Heliyon. 2020;6(4):e03690. doi:10.1016/j.heliyon.2020.e03690. 32322707 PMC7160433

[3] Kotloff KL, Platts-Mills JA, Nasrin D, Roose A, Blackwelder WC, Levine MM. Global burden of diarrheal diseases among children in developing countries: incidence, etiology, and insights from new molecular diagnostic techniques. Vaccine. 2017;35(49):6783–6789. doi:10.1016/j.vaccine.2017.07.036. 28765005

[4] Jesudason T, Rodarte A, Tordrup D, Carias C, Chen Y-H. Systematic review of rotavirus vaccination cost-effectiveness in high income settings utilising dynamic transmission modelling techniques. Vaccine. 2023;41(36):5221–5232. doi:10.1016/j.vaccine.2023.06.064. 37479614

[5] Lewnard JA, McQuade ETR, Platts-Mills JA, Kotloff KL, Laxminarayan R. Incidence and etiology of clinically-attended, antibiotic-treated diarrhea among children under five years of age in low-and middle-income countries: evidence from the global enteric multicenter study. PLOS Negl Trop Dis. 2020;14(8):1–21. doi:10.1371/journal.pntd.0008520. PMC7444547 32776938

[6] Operario DJ, Platts-Mills JA, Nadan S, Page N, Seheri M, Mphahlele J, Praharaj I, Kang G, Araujo IT, Leite JPG, et al. Etiology of severe acute watery diarrhea in children in the global rotavirus surveillance network using quantitative polymerase chain reaction. J Infect Dis. 2017;216(2):220–227. doi:10.1093/infdis/jix294. 28838152 PMC5853801

[7] Florez ID, Niño-Serna LF, Beltrán-Arroyave CP. Acute infectious diarrhea and gastroenteritis in children. Curr Infect Dis Rep. 2020 22;22(2). doi:10.1007/s11908-020-0713-6. 31993758

[8] Rivera-Dominguez G, Ward R. Pediatric gastroenteritis - StatPearls - NCBI bookshelf. Treasure Island (FL), USA: StatPearls Publishing; 2023 [accessed 2023 Apr 8]. https://www.ncbi.nlm.nih.gov/books/NBK499939/.

[9] Flynn TG, Olortegui MP, Kosek MN. Viral gastroenteritis. Lancet. 2024;403(10429):862–876. doi:10.1016/S0140-6736(23)02037-8. 38340741

[10] Schierenberg A, Bruijning-Verhagen PCJ, Van Delft S, Bonten MJM, De Wit NJ. Antibiotic treatment of gastroenteritis in primary care. J Antimicrob Chemother. 2019;74:207–213. doi:10.1093/jac/dky385. 30285243

[11] Brennhofer SA, Platts-Mills JA, Lewnard JA, Liu J, Houpt ER, Rogawski McQuade ET. Antibiotic use attributable to specific aetiologies of diarrhoea in children under 2 years of age in low-resource settings: a secondary analysis of the MAL-ED birth cohort. BMJ Open. 2022;12(4):1–9. doi:10.1136/bmjopen-2021-058740. PMC8977746 35365541

[12] Rhee C, Aol G, Ouma A, Audi A, Muema S, Auko J, Omore R, Odongo G, Wiegand RE, Montgomery JM, et al. Inappropriate use of antibiotics for childhood diarrhea case management — Kenya, 2009–2016. BMC Public Health. 2019;19(S3):1–12. doi:10.1186/s12889-019-6771-8. 32326936 PMC6696675

[13] Efunshile AM, Ezeanosike O, Nwangwu CC, König B, Jokelainen P, Robertson LJ. Apparent overuse of antibiotics in the management of watery diarrhoea in children in Abakaliki, Nigeria. BMC Infect Dis. 2019;19(1):1–7. doi:10.1186/s12879-019-3899-1. 30898105 PMC6429783

[14] Okubo Y, Miyairi I, Michihata N, Morisaki N, Kinoshita N, Urayama KY, Yasunaga H. Recent prescription patterns for children with acute infectious diarrhea. J Pediatr Gastroenterol Nutr. 2019;68(1):13–16. doi:10.1097/MPG.0000000000002115. 30074577

[15] Lewnard JA, Lo NC, Arinaminpathy N, Frost I, Laxminarayan R. Childhood vaccines and antibiotic use in low- and middle-income countries. Nature. 2020;581(7806):94–99. 10.1038/s41586-020-2238-4. 32376956 PMC7332418

[16] Clift C, Salisbury DM. Enhancing the role of vaccines in combatting antimicrobial resistance. Vaccine. 2017;35(48):6591–6593. doi:10.1016/j.vaccine.2017.09.053. 29153150 PMC5714609

[17] Obolski U, Lourenço J, Thompson C, Thompson R, Gori A, Gupta S. Vaccination can drive an increase in frequencies of antibiotic resistance among nonvaccine serotypes of Streptococcus pneumoniae. Proc Natl Acad Sci USA. 2018;115(12):3102–3107. doi:10.1073/pnas.1718712115. 29511100 PMC5866575

[18] Laxminarayan R, Duse A, Wattal C, Zaidi AKM, Wertheim HFL, Sumpradit N, Vlieghe E, Hara GL, Gould IM, Goossens H, et al. Antibiotic resistance—the need for global solutions. Lancet Infect Dis. 2013;13(12):1057–1098. doi:10.1016/S1473-3099(13)70318-9. 24252483

[19] Doherty TM, Hausdorff WP, Kristinsson KG. Effect of vaccination on the use of antimicrobial agents: a systematic literature review. Ann Med. 2020;52(6):283–299. doi:10.1080/07853890.2020.1782460. 32597236 PMC7880080

[20] Ginsburg AS, Klugman KP. Vaccination to reduce antimicrobial resistance. Lancet Glob Health. 2017;5(12):e1176–e1177. doi:10.1016/S2214-109X(17)30364-9. 29128252

[21] Klugman KP, Black S. Impact of existing vaccines in reducing antibiotic resistance: primary and secondary effects. Proc Natl Acad Sci USA. 2018;115(51):12896–12901. doi:10.1073/pnas.1721095115. 30559195 PMC6304973

[22] Buckley BS, Henschke N, Bergman H, Skidmore B, Klemm EJ, Villanueva G, Garritty C, Paul M. Impact of vaccination on antibiotic usage: a systematic review and meta-analysis. Clin Microbiol Infect. 2019;25(10):1213–1225. doi:10.1016/j.cmi.2019.06.030. 31284031

[23] Knight GM, Clarkson M, De Silva TI. Potential impact of influenza vaccine roll-out on antibiotic use in Africa. J Antimicrob Chemother. 2018;73(8):2197–2200. doi:10.1093/jac/dky172. 29746637 PMC6054263

[24] Vesikari T, Matson DO, Dennehy P, Van Damme P, Santosham M, Rodriguez Z, Dallas MJ, Heyse JF, Goveia MG, Black SB, et al. Safety and efficacy of a pentavalent human–bovine (WC3) reassortant rotavirus vaccine. N Engl J Med. 2006;354(1):23–33. doi:10.1056/nejmoa052664. 16394299

[25] Armah GE, Sow SO, Breiman RF, Dallas MJ, Tapia MD, Feikin DR, Binka FN, Steele AD, Laserson KF, Ansah NA, et al. Efficacy of pentavalent rotavirus vaccine against severe rotavirus gastroenteritis in infants in developing countries in sub-Saharan Africa: a randomised, double-blind, placebo-controlled trial. Lancet. 2010;376(9741):606–614. doi:10.1016/S0140-6736(10)60889-6. 20692030

[26] Ruiz-Palacios GM, Pérez-Schael I, Velázquez FR, Abate H, Breuer T, Clemens SC, Cheuvart B, Espinoza F, Gillard P, Innis BL, et al. Safety and efficacy of an attenuated vaccine against severe rotavirus gastroenteritis. N Engl J Med. 2006;354(1):11–22. doi:10.1056/NEJMoa052434. 16394298

[27] Bhandari N, Rongsen-Chandola T, Bavdekar A, John J, Antony K, Taneja S, Goyal N, Kawade A, Kang G, Rathore SS, et al. Efficacy of a monovalent human-bovine (116E) rotavirus vaccine in Indian infants: a randomised, double-blind, placebo-controlled trial. Lancet. 2014;383(9935):2136–2143. doi:10.1016/S0140-6736(13)62630-6. 24629994 PMC4532697

[28] Muhsen K, Omar M. Rotavirus. In: Tang YW, Hindiyeh MY, Liu D, Sails A, Spearman P, Zhang JR, editors. Molecular medical microbiology. Academic Press; 2024. p. 2321–2338. doi:10.1016/B978-0-12-818619-0.00052-6.

[29] Varghese T, Kang G, Steele AD. Understanding rotavirus vaccine efficacy and effectiveness in countries with high child mortality. Vaccines(Basel). 2022 10;10(3):346. doi:10.3390/vaccines10030346. 35334978 PMC8948967

[30] Glass RI, Tate JE, Jiang B, Parashar U. The rotavirus vaccine story: from discovery to the eventual control of rotavirus disease. J Infect Dis. 2021;224(Supplement_4):S331–S342. doi:10.1093/infdis/jiaa598. 34590142 PMC8482027

[31] Kulkarni PS, Desai S, Tewari T, Kawade A, Goyal N, Garg BS, Kumar D, Kanungo S, Kamat V, Kang G, et al. A randomized phase III clinical trial to assess the efficacy of a bovine-human reassortant pentavalent rotavirus vaccine in Indian infants. Vaccine. 2017;35(45):6228–6237. doi:10.1016/j.vaccine.2017.09.014. 28967523 PMC5651219

[32] Burnett E, Parashar UD, Tate JE. Malnourished children: a review of the literature. 2021;XX:1–7. doi:10.1097/INF.0000000000003206. PMC8489158 34117200

[33] Thomas SL, Walker JL, Fenty J, Atkins KE, Elliot AJ, Hughes HE, Stowe J, Ladhani S, Andrews NJ. Impact of the national rotavirus vaccination programme on acute gastroenteritis in England and associated costs averted. Vaccine. 2017;35(4):680–686. doi:10.1016/j.vaccine.2016.11.057. 28007397 PMC5267482

[34] Aliabadi N, Antoni S, Mwenda JM, Weldegebriel G, Biey JNM, Cheikh D, Fahmy K, Teleb N, Heffelfinger JD, Fox K, et al. Global impact of rotavirus vaccine introduction on rotavirus hospitalisations among children under 5 years of age, 2008–16: findings from the global rotavirus surveillance network. Lancet Global Health. 2019;7(7):893–903. doi:10.1016/S2214-109X(19)30207-4. PMC7336990 31200889

[35] Velázquez RF, Linhares AC, Muñoz S, Seron P, Lorca P, DeAntonio R, Ortega-Barria E. Efficacy, safety and effectiveness of licensed rotavirus vaccines: a systematic review and meta-analysis for Latin America and the Caribbean. BMC Pediatr. 2017;17(1):1–12. doi:10.1186/s12887-016-0771-y. 28086819 PMC5237165

[36] Muhsen K, Chodick G, Goren S, Anis E, Ziv-Baran T, Shalev V, Cohen D. Change in incidence of clinic visits for all-cause and rotavirus gastroenteritis in young children following the introduction of universal rotavirus vaccination in Israel. Eurosurveillance. 2015;20(42):1–9. doi:10.2807/1560-7917.ES.2015.20.42.30045. 26538450

[37] Muhsen K, Anis E, Rubinstein U, Kassem E, Goren S, Shulman LM, Ephros M, Cohen D. Effectiveness of rotavirus pentavalent vaccine under a universal immunization programme in Israel, 2011–2015: a case–control study. Clin Microbiol Infect. 2018;24(1):53–59. doi:10.1016/j.cmi.2017.04.018. 28442435

[38] Muhsen K, Rubenstein U, Kassem E, Goren S, Schachter Y, Kremer A, Shulman LM, Ephros M, Cohen D. A significant and consistent reduction in rotavirus gastroenteritis hospitalization of children under 5 years of age, following the introduction of universal rotavirus immunization in Israel. Hum Vaccin Immunother. 2015;11(10):2475–2482. doi:10.1080/21645515.2015.1056951. 26212174 PMC4635902

[39] Hall EW, Tippett A, Fridkin S, Anderson EJ, Lopman B, Benkeser D, Baker JM. Association between rotavirus vaccination and antibiotic prescribing among commercially insured US children, 2007–2018. Open Forum Infect Dis. 2022;9(7). doi:10.1093/ofid/ofac276. PMC9291383 35855006

[40] The Organization for Economic Co-operation and Development (OECD), country statistical profile Israel. n.d [accessed 2024 Feb 11]. https://data.oecd.org/israel.htm.

[41] Omar M, Kassem E, Abu-Jabal R, Mwassi B, Cohen D, Muhsen K. Characterization of antibiotic treatment among children aged 0–59 months hospitalized for acute bacterial gastroenteritis in Israel. Antibiotics. 2024 13;13(1):64. doi:10.3390/antibiotics13010064. 38247623 PMC10812600

[42] Central Bureau of Statistics. Statistical Abstract of Israel 2008. Publication number 59. Jerusalem: State of Israel; 2008 [accessed 2009 June 14]. http://www1.cbs.gov.il/reader/shnatonhnew_site.htm.

[43] Israel Central Bureau of Statistics. Statistical abstract of Israel 2015. Publication number 66; Jerusalem: State of Israel; 2016 [accessed 2015 Sep 10]. https://www.cbs.gov.il/en/publications/Pages/2015/Statistical-Abstract-of-Israel-2015-No66.aspx.

[44] Porath AL. The new Israeli national health insurance law and quality of care. Int J Qual Health Care. 1995;7(3):281–284. doi:10.1093/intqhc/7.3.281. 8595467

[45] Elran B, Yaari S, Glazer Y, Honovich M, Grotto I, Anis E. Parents’ perceptions of childhood immunization in Israel: information and concerns. Vaccine. 2018;36:8062–8068. doi:10.1016/j.vaccine.2018.10.078. 30473184

[46] Muhsen K, Cohen D. Rotavirus vaccines in Israel: uptake and impact. Hum Vaccin Immunother. 2017;13(7):1722–1727. doi:10.1080/21645515.2017.1297908. 28281866 PMC5512754

[47] Muhsen K, Haklai Z, Applbaum Y, Gordon E-S, Shteiman A, Glatman-Freedman A, Leshem E. Effects of rotavirus vaccine on all-cause acute gastroenteritis and rotavirus hospitalizations in Israel: a nationwide analysis. Vaccine. 2020;38(10):2406–2415. doi:10.1016/j.vaccine.2020.01.034. 32029322

[48] Muhsen K, Kassem E, Rubenstein U, Goren S, Ephros M, Cohen D, Shulman LM. Incidence of rotavirus gastroenteritis hospitalizations and genotypes, before and five years after introducing universal immunization in Israel. Vaccine. 2016;34(48):5916–5922. doi:10.1016/j.vaccine.2016.10.021. 27771186

[49] King LM, Bartoces M, Fleming-Dutra KE, Roberts RM, Hicks LA. Changes in US outpatient antibiotic prescriptions from 2011-2016. Clin Infect Dis. 2020;70:370–377. doi:10.1093/cid/ciz225. 30882145 PMC8078491

[50] Palin V, Mölter A, Belmonte M, Ashcroft DM, White A, Welfare W, van Staa T. Antibiotic prescribing for common infections in UK general practice: variability and drivers. J Antimicrob Chemother. 2019;74(8):2440–2450. doi:10.1093/jac/dkz163. 31038162 PMC6640319

[51] Muhsen K, Shulman L, Rubinstein U, Kasem E, Kremer A, Goren S, Zilberstein I, Chodick G, Ephros M, Cohen D. Incidence, characteristics, and economic burden of rotavirus gastroenteritis associated with hospitalization of Israeli children <5 years of age, 2007–2008. J Infect Dis. 2009;200:S254–S263. doi:10.1086/605425. 19817606

[52] Muhsen K, Kassem E, Rubenstein U, Goren S, Ephros M, Shulman LM, Cohen D. No evidence of an increase in the incidence of norovirus gastroenteritis hospitalizations in young children after the introduction of universal rotavirus immunization in Israel. Hum Vaccines Immunother. 2019;15(6):1284–1293. doi:10.1080/21645515.2019.1599522. PMC6663133 30945960

[53] Stein-Zamir C, Zentner G, Tallen-Gozani E, Grotto I, Gamzu R. The national childhood immunization registry in Israel. Procedia Vaccinol. 2011;4:9–13. doi:10.1016/j.provac.2011.07.002.

[54] Stein-Zamir C, Zentner G, Fracp MB, Tallen-Gozani E, Grotto I. The Israel national immunization registry. 2010. 20929084

[55] da Cruz Gouveia MA, Lins MTC, da Silva GAP. Acute diarrhea with blood: diagnosis and drug treatment. J Pediatr (Rio J). 2020;96:20–28. doi:10.1016/j.jped.2019.08.006. PMC9432323 31604059

[56] Sattar SBA, Singh S. Bacterial gastroenteritis. StatPearls. Treasure Island (FL), USA: StatPearls Publishing; 2023. [accessed 2023 Aug 8]. https://www.ncbi.nlm.nih.gov/books/NBK513295/.

[57] Phillips G, Lopman B, Tam CC, Iturriza-Gomara M, Brown D, Gray J. Diagnosing norovirus-associated infectious intestinal disease using viral load. BMC Infect Dis. 2009;9(1):63. doi:10.1186/1471-2334-9-63. 19442278 PMC2698835

[58] Kageyama T, Kojima S, Shinohara M, Uchida K, Fukushi S, Hoshino FB, Takeda N, Katayama K. Broadly reactive and highly sensitive assay for Norwalk-like viruses based on real-time quantitative reverse transcription-pcr. J Clin Microbiol. 2003;41(4):1548–1557. doi:10.1128/JCM.41.4.1548-1557.2003. 12682144 PMC153860

[59] Liu J, Platts-Mills JA, Juma J, Kabir F, Nkeze J, Okoi C, Operario DJ, Uddin J, Ahmed S, Alonso PL, et al. Use of quantitative molecular diagnostic methods to identify causes of diarrhoea in children: a reanalysis of the GEMS case-control study. Lancet. 2016;388(10051):1291–1301. doi:10.1016/S0140-6736(16)31529-X. 27673470 PMC5471845

[60] Trang NV, Choisy M, Nakagomit, Chinh NTM, Doan YH, Yamashiro T, Nakagomi T, Bryant JE, Nakagomi O, Anh DD. Determination of cut-off cycle threshold values in routine RT–PCR assays to assist differential diagnosis of norovirus in children hospitalized for acute gastroenteritis. Epidemiol Infect. 2015;143(15):3292–3299. doi:10.1017/S095026881500059X. 26418350 PMC4594052

[61] Phillips G, Tam CC, Conti S, Rodrigues LC, Brown D, Iturriza-Gomara M, Gray J, Lopman B. Community incidence of norovirus-associated infectious intestinal disease in England: improved estimates using viral load for norovirus diagnosis. Am J Epidemiol. 2010;171(9):1014–1022. doi:10.1093/aje/kwq021. 20360244

[62] Brownlee KA. Statistical theory and methodology in science and engineering; a Wiley publication in applied statistics. Hoboken (NJ), USA: John Wiley & Sons; 1965.

[63] Korppi M, Kröger L. C-reactive protein in viral and bacterial respiratory infection in children. Scand J Infect Dis. 1993;25(2):207–213. doi:10.3109/00365549309008486. 8511515

[64] Abramson JH. WINPEPI updated: computer programs for epidemiologists, and their teaching potential. Epidemiologic Perspect & Innovations. 2011;8(1):1. Available on: http://www.ncbi.nlm.nih.gov/pmc/articles/PMC3041648/pdf/1742-5573-8-1.pdf. 10.1186/1742-5573-8-1 PMC3041648 21288353

[65] Rinawi F, As WM, Se SR. Recommendations for the diagnosis and management of pediatric acute gastroenteritis in Israel - Update 2017. Harefuah. 2017;156(3):189–93. 28551933

[66] Kim YJ, Park KH, Park DA, Park J, Bang BW, Lee SS, Lee EJ, Lee HJ, Hong SK, Kim YR. Guideline for the antibiotic use in acute gastroenteritis. Infect Chemother. 2019;51(2):217–243. doi:10.3947/ic.2019.51.2.217. 31271003 PMC6609748

[67] Sansonetti P, Bergounioux J. Shigellosis. In: Loscalzo J, Fauci A, Kasper D, Hauser S, Longo D, Jameson J, editors. Harrison’s Principles of Internal Medicine. Vol. 1. 21st ed. (NY), NY, USA: McGraw Hill; 2022. p. 1298–1130.

[68] Blaser MJ. Infections due to Campylobacter and related organisms. In: Loscalzo J, Fauci A, Kasper D, Hauser S, Longo D, Jameson J, editors. Harrison’s Principles of Internal Medicine. Vol. 1. 21st ed. New York, NY, USA: McGraw Hill; 2022. p. 1302–1304.

[69] Pegues DA. MSI Salmonellosis. In: Loscalzo J, Fauci A, Kasper D, Hauser S, Longo D, Jameson J, editors. Harrison’s Principles of Internal Medicine. Vol. 1. 21st ed. New York, NY, USA: McGraw Hill; 2022. p. 1291–1297.

[70] Akhavan BK. Amoxicillin. StatPearls—NCBI Bookshelf. StatPearls. Treasure Island (FL), USA: StatPearls Publishing; 2023 [accessed 2023 Nov 17]. https://www.ncbi.nlm.nih.gov/books/NBK482250/.

[71] Evans JH. Amoxicillin clavulanate. StatPearls—NCBI Bookshelf. StatPearls. Treasure Island (FL), USA: StatPearls Publishing; 2023 [accessed 2023 Aug 16]. https://www.ncbi.nlm.nih.gov/books/NBK538164/.

[72] Troeger C, Khalil IA, Rao PC, Cao S, Blacker BF, Ahmed T, Armah G, Bines JE, Brewer TG, Colombara DV, et al. Rotavirus vaccination and the global burden of rotavirus diarrhea among children younger than 5 years. JAMA Pediatr. 2018;172(10):958–965. doi:10.1001/jamapediatrics.2018.1960. 30105384 PMC6233802

[73] Smith ER, Fry AM, Hicks LA, Fleming-Dutra KE, Flannery B, Ferdinands J, Rolfes MA, Martin ET, Monto AS, Zimmerman RK, et al. Reducing antibiotic use in ambulatory care through influenza vaccination. Clin Infect Dis. 2020;71(11):726–734. doi:10.1093/cid/ciaa464. PMC7778345 32322875

[74] Klein EY, Schueller E, Tseng KK, Morgan DJ, Laxminarayan R, Nandi A. The Impact of Influenza Vaccination on Antibiotic Use in the United States, 2010–2017. Open Forum Infect Dis. 2020;7(7):7. doi:10.1093/ofid/ofaa223. PMC7336555 32665959

[75] Nichol KL, Mendelman PM, Mallon KP, Jackson LA, Gorse GJ, Belshe RB, Glezen WP. Intranasal influenza virus vaccine. Vaccine. 1999;282(2):137–144. doi:10.1001/jama.282.2.137. 10411194

[76] Kwong JC, Maaten S, Upshur REG, Patrick DM, Marra F. The effect of universal influenza immunization on antibiotic prescriptions: An ecological study. Clin Infect Dis. 2009;49(5):750–756. doi:10.1086/605087. 19624280

[77] Wi D, Choi SH. Antibiotic prescribing practices and clinical outcomes of pediatric patients with Campylobacter enterocolitis. Children. 2023;10(1):1–10. doi:10.3390/children10010040. PMC9856514 36670591

[78] Vecchio AL, Liguoro I, Bruzzese D, Scotto R, Parola L, Gargantini G, Guarino A. Adherence to guidelines for management of children hospitalized for acute diarrhea. Pediatr Infect Disease J. 2014;33(11):1103–1108. doi:10.1097/INF.0000000000000396. 24830697

[79] Obolski U, Kassem E, Na’amnih W, Tannous S, Kagan V, Muhsen K. Unnecessary antibiotic treatment of children hospitalised with respiratory syncytial virus (RSV) bronchiolitis: risk factors and prescription patterns. J Glob Antimicrob Resist. 2021;27:303–308. doi:10.1016/j.jgar.2021.10.015. 34718202

