# Supplementary material for: Factors associated with antibiotic use in children hospitalized for acute viral gastroenteritis and the relation to rotavirus vaccination
Source: Hum Vaccin Immunother. 2024 Sep 9;20(1):2396707. doi: 10.1080/21645515.2024.2396707 (PMC11385160; doi:10.1080/21645515.2024.2396707)
Supplement: Supplementary material final.docx [file KHVI_A_2396707_SM5059.docx]

**Supplementary Table 1: Bacterial co-infections with AGE**

|  | **N** | **%** |
| --- | --- | --- |
| **Any bacterial co-infections with AGE** | 251 | 11.2% |
| Pneumonia | 76 | 3.0% |
| Bacteremia | 11 | 0.4% |
| Urinary tract infection | 55 | 21.9% |
| Tonsilitis | 10 | 4.0% |
| Otitis media | 94 | 37.5% |
| Lymphadenitis | 4 | 1.6% |
| Impetigo | 2 | 0.8% |
| Cellulitis | 6 | 2.4% |
| Osteomyelitis | 2 | 0.8% |
| Mastoiditis | 1 | 0.4% |

*Children with AGE could have one bacterial infection or more, therefore the percentage exceeds 100%. AGE= Acute gastroenteritis.

**Supplementary Table 2: Hematology and biochemistry tests of children aged 0-59 months hospitalized for acute gastroenteritis.**

| **Tests** | **N** | **Min** | **Max** | **Median** | **IQR** | **Mean** | **SD** |
| --- | --- | --- | --- | --- | --- | --- | --- |
| Hemoglobin (g/dl) | 2135 | 7.1 | 28.4 | 11.6 | 1.6 | 11.6 | 1.4 |
| Platelets (K/µl) | 2135 | 19.0* | 1063.0 | 357.0 | 158.9 | 376.2 | 131.2 |
| Leukocytes (K/µl) | 2139 | 2.4 | 48.8 | 12.3 | 7.3 | 13.5 | 6.1 |
| Neutrophils (K/µl) | 2132 | 0.1 | 86.1 | 6.4 | 6.1 | 7.6 | 5.4 |
| Lymphocytes (K/µl) | 2133 | 0.3 | 44.7 | 4.1 | 3.5 | 4.5 | 3.0 |
| Glucose (mg/dL) | 2108 | 30.0 | 273.0 | 90.0 | 24.0 | 91.3 | 23.2 |
| Blood urea nitrogen, mg/dL | 2127 | 0.3 | 55.7 | 11.0 | 7.0 | 11.8 | 5.9 |
| Creatinine (mg/dL) | 2071 | 0.03 | 2.2 | 0.3 | 0.1 | 0.3 | 0.1 |
| C-reactive protein (mg/L) | 1984 | 0.0 | 369.0 | 11.9 | 37.9 | 32.7 | 49.6 |
| Sodium mEq/l | 2128 | 121.1 | 189.5 | 137.8 | 4.0 | 137.9 | 3.9 |
| Potassium mEq/l | 1946 | 2.3 | 8.0 | 4.5 | 0.7 | 4.6 | 0.6 |

IQR= Interquartile range; Max= Maximum; Min= Minimum; SD=Standard deviation. *Three children had thrombocytopenia with platelets <50 (K/µl), other values are above> 50 (K/µl).

**Supplementary Table 3: Classification of infections of children hospitalized for AGE**

| **Classification of infections** | **N** | **%** |
| --- | --- | --- |
| Total infections | 2240 | 100% |
| Culture-proven bacterial AGE or dysentery | 550 | 24.5% |
| Bacterial co-infections alone | 219 | 9.8% |
| Likely bacterial infections alone | 58 | 2.6% |
| Viral infections | 1395 | 62.3% |
| Other conditions | 18 | 0.8% |

AGE= Acute gastroenteritis.

**Supplementary Table 4: Demographic and clinical factors associated with unnecessary antibiotic use among children hospitalized with viral AGE and CRP ≤ 50 mg/L - Bivariate analysis**

| **P value** | **No antibiotic treatment**  **during hospitalization N= 1135** | **Antibiotic treatment during hospitalization**  **N=141** |  |
| --- | --- | --- | --- |
| 0.077 |  |  | Age (months), N (%) |
|  | 505 (44.5%) | 76 (53.9%) | 0-11 |
|  | 365 (32.2%) | 34 (24.1%) | 12-23 |
|  | 265 (23.3%) | 31 (22.0%) | 24-59 |
| 0.401 | 614 (54.1%) | 71 (50.4%) | Sex, males, N (%) |
| 0.411 | 581 (51.2%) | 67 (47.5%) | Ethnicity (Jewish vs. Arabs), N (%) |
| 0.320 |  |  | Residential socioeconomic status rank, N (%) |
|  | 456 (43.4%) | 61 (44.9%) | 1-3 (Low) |
|  | 419 (39.9%) | 59 (43.4%) | 4-5 (Intermediate) |
|  | 176 (16.7%) | 16 (11.8%) | 6-10 (High) |
| <0.001 |  |  | Year of admission, N (%) |
|  | 54 (4.8%) | 1 (0.7%) | 2008 |
|  | 119 (10.5%) | 8 (5.7%) | 2009 |
|  | 259 (22.8%) | 21 (14.9%) | 2010 |
|  | 105 (9.3%) | 10 (7.1%) | 2011 |
|  | 207 (18.2%) | 44 (31.2%) | 2012 |
|  | 182 (16.0%) | 16 (11.3%) | 2013 |
|  | 138 (12.2%) | 26 (18.4%) | 2014 |
|  | 71 (6.3%) | 15 (10.6%) | 2015 |
| 0.010 | 187 (16.5%) | 28 (19.9%) | Background diseases, N (%) |
| 0.004 | 451 (39.7%) | 74 (52.5%) | Previous lifetime hospitalization, N (%) |
| 0.138 | 178 (15.7%) | 29 (20.6%) | Previous AGE hospitalization, N (%) |
| 0.024 | 379 (33.7%) | 61 (43.3%) | Fever on admission, N (%) |
| <0.001 | 1001 (88.2%) | 110 (78.0%) | Vomiting, N (%) |
| 0.124 |  |  | Number of stools on the severe day, N (%) |
|  | 518 (45.6%) | 74 (52.5%) | 0-5 |
|  | 617 (54.4%) | 67 (45.5%) | ≥6 |
| <0.001 | 386 (81.3%) | 56 (64.4%) | Receiving rotavirus vaccine*, N (%) |
| <0.001 | 64 (5.6%) | 41 (29.1%) | Chest-X-ray test performed, N (%) |
| <0.001 | 335 (29.5%) | 74 (52.5%) | Blood culture performed, N (%) |
| <0.001 | 38 (3.3%) | 36 (25.5%) | Urine culture performed, N (%) |
| 0.976 | 723 (63.7%) | 90 (63.8%) | Rotavirus test performed, N (%) |
| <0.001 |  |  | Rotavirus test results^§^, N (%) |
|  | 253 (35.0%) | 18 (20.0%) | Positive |
|  | 410 (56.7%) | 70 (77.8%) | Negative |
|  | 60 (8.3%) | 2 (2.2%) | Unknown |
| 0.390 | 11.8 (1.4), N=1064 | 11.7 (1.4), N=138 | Hemoglobin (g/dl), mean (SD)^#^ |
| <0.001 | 378.7 (129.3), N=1065 | 420.1 (173.3), N=138 | Platelets (K/µl), mean (SD) ^#^ |
| <0.001 | 12.5 (5.3), N=1066 | 14.8 (7.8), N=138 | Leukocytes (K/µl), mean (SD) ^#^ |
| 0.004 | 6.9 (4.6), N=1064 | 8.2 (5.9), N=137 | Neutrophils (K/µl), mean (SD) ^#^ |
| 0.088 | 4.3 (2.8), N=1063 | 4.7 (2.6), N=138 | Lymphocytes(K/µl), mean (SD) ^#^ |
| 0.005 | 86.7 (22.8), N=1055 | 92.6 (20.1), N=130 | Glucose (mg/dL), mean (SD) ^#^ |
| 0.056 | 13.1 (5.9), N=1063 | 12.0 (7.3), N=133 | Blood urea nitrogen(mg/dL), mean (SD) ^#^ |
| 0.435 | 0.3 (0.2), N=1031 | 0.3 (0.2), N=131 | Creatinine(mg/dL), mean (SD) ^#^ |
| <0.001 | 5.3 (12.3), N=982 | 9.3 (16.5), N=108 | C-reactive protein (mg/L), median (IQR) ^#^ |
| 0.440 | 138.4 (3.7), N=1062 | 138.7 (5.2), N=133 | Sodium mEq/l, mean (SD) ^#^ |
| 0.005 | 4.6 (0.6), N=973 | 4.7 (0.8), N=119 | Potassium mEq/l, mean (SD) ^#^ |

*This analysis is based on children with viral AGE eligible for rotavirus vaccination in the universal vaccination program – birth cohorts 2011-2015. ^§^ This analysis included only children who performed rotavirus test. ^#^ The numbers (N) represent participants with available data. AGE= Acute gastroenteritis; IQR= Interquartile range; SD=Standard deviation.

**Supplementary Table 5: Demographic and clinical factors associated with unnecessary antibiotic use in children hospitalized with viral AGE - who belong to cohort birth 2012-2015, Bivariate analysis**

| **P value** | **No antibiotic treatment**  **during hospitalization N= 343** | **Antibiotic treatment during hospitalization**  **N=80** |  |
| --- | --- | --- | --- |
| 0.187 |  |  | Age (months), N (%) |
|  | 227 (66.2%) | 53 (66.3%) | 0-11 |
|  | 102 (29.7%) | 20 (25%) | 12-23 |
|  | 14 (4.1%) | 7 (8.8%) | 24-59 |
| 0.170 | 192 (56.0%) | 38 (47.5%) | Sex, males, N (%) |
| 0.025 | 185 (53.9%) | 32 (40.0%) | Ethnicity (Jewish vs. Arabs), N (%) |
| 0.430 |  |  | Residential socioeconomic status rank, N (%) |
|  | 133 (41.6%) | 37 (48.1%) | 1-3 (Low) |
|  | 133 (41.6%) | 31 (40.3%) | 4-5 (Intermediate) |
|  | 54 (16.9%) | 9 (11.7%) | 6-10 (High) |
| 0.289 |  |  | Year of admission, N (%) |
|  | 44 (12.8%) | 13 (16.3%) | 2012 |
|  | 115 (33.5%) | 18 (22.5%) | 2013 |
|  | 116 (33.8%) | 31 (38.8%) | 2014 |
|  | 68 (19.8%) | 18 (22.5%) | 2015 |
| 0.028 | 44 (12.8%) | 18 (22.5%) | Background diseases, N (%) |
| 0.051 | 139 (40.5%) | 42 (52.5%) | Previous lifetime hospitalization, N (%) |
| 0.710 | 46 (13.4%) | 12 (15.0%) | Previous AGE hospitalization, N (%) |
| 0.006 | 131 (38.2%) | 44 (55.0%) | Fever on admission, N (%) |
| 0.040 | 276 (80.5%) | 56 (70.0%) | Vomiting, N (%) |
| 0.742 |  |  | Number of stools on the severe day, N (%) |
|  | 194 (56.7%) | 47 (58.8%) | 0-5 |
|  | 148 (43.3%) | 33 (41.3%) | ≥6 |
| <0.001 | 61 (18.1%) | 29 (37.7%) | Received rotavirus vaccine*, N (%) |
| <0.001 | 26 (7.6%) | 29 (36.3%) | Chest-X-ray test performed, N (%) |
| 0.002 | 222 (64.7%) | 66 (82.5%) | Blood culture performed, N (%) |
| <0.001 | 21 (6.1%) | 28 (35.0%) | Urine culture performed, N (%) |
| 0.640 | 207 (60.3%) | 46 (57.5%) | Rotavirus test performed, N (%) |
| 0.178 |  |  | Rotavirus test results^§^, N (%) |
|  | 35 (16.9%) | 3 (6.5%) | Positive |
|  | 171 (82.6%) | 43 (93.5%) | Negative |
|  | 1 (0.5%) | 0 (0.0%) | Unknown |
| 0.803 | 11.4 (1.4), N=323 | 11.4 (1.4), N=79 | Hemoglobin (g/dl), mean (SD)^#^ |
| 0.012 | 378.9 (132.8), N=323 | 424.6 (181.7), N=79 | Platelets (K/µl), mean (SD) ^#^ |
| <0.001 | 12.5 (4.9), N=323 | 14.8 (7.3), N=79 | Leukocytes (K/µl), mean (SD) ^#^ |
| 0.015 | 5.9 (4.0), N=321 | 7.2 (4.8), N=77 | Neutrophils (K/µl), mean (SD) ^#^ |
| 0.897 | 5.2 (2.5), N=321 | 5.2 (2.5), N=78 | Lymphocytes(K/µl), mean (SD) ^#^ |
| 0.001 | 89.3 (20.0), N=324 | 97.7 (20.4), N=72 | Glucose (mg/dL), mean (SD) ^#^ |
| 0.562 | 11.5 (5.2), N=326 | 11.0 (7.8), N=75 | Blood urea nitrogen(mg/dL), mean (SD) ^#^ |
| 0.055 | 0.3 (0.1), N=315 | 0.3 (0.2), N=73 | Creatinine(mg/dL), mean (SD) ^#^ |
| <0.001 | 6.6 (14.9), N=301 | 18.9 (49.5), N=66 | C-reactive protein (mg/L), median (IQR) ^#^ |
| 0.068 | 138.0 (3.4), N=326 | 138.9 (5.7), N=74 | Sodium mEq/l, mean (SD) ^#^ |
| 0.244 | 4.7 (0.6), N=312 | 4.8 (0.8), N=67 | Potassium mEq/l, mean (SD) ^#^ |

^§^ This analysis included only children who performed rotavirus test. ^#^ The numbers (N) represent participants with available data. AGE= Acute gastroenteritis; IQR= Interquartile range; SD=Standard deviation.

**Supplementary Table 6: Logistic regression analysis of factors associated with unnecessary antibiotic use during and after hospitalizations in children with viral AGE eligible for receiving rotavirus vaccination (birth cohort 2012-2015) during the universal vaccination years**

| **P value** | **Adjusted OR (95% CI)** | **P value** | **Unadjusted OR (95% CI)** | **Variable** |
| --- | --- | --- | --- | --- |
| 0.055 | 1.07 (1.00-1.14) | 0.001 | 1.07 (1.03-1.12) | Leukocytes (K/µl), continuous variable |
| <0.001 | 1.03 (1.01-1.04) | <0.001 | 1.03 (1.02-1.04) | C-reactive protein (mg/L), continuous variable |
| 0.009 | 0.33 (0.15-0.76) | <0.001 | 0.37 (0.21-0.63) | Rotavirus vaccination, (yes, vs. no) |
| 0.012 |  | 0.297 |  | **Year of admission** |
|  | Reference |  | Reference | 2012 |
| 0.001 | 0.16 (0.05-0.49) | 0.117 | 0.53 (0.24-1.17) | 2013 |
| 0.005 | 0.21 (0.07-0.62) | 0.789 | 0.91 (0.43-1.89) | 2014 |
| 0.033 | 0.27 (0.08-0.90) | 0.790 | 0.89 (0.40-2.01) | 2015 |
| 0.009 | 3.22 (1.34-7.74) | <0.001 | 6.93 (3.78-12.71) | Chest X-ray test performed, yes vs. no |
| 0.048 | 2.66 (1.01-7.04) | 0.003 | 2.57 (1.39-4.77) | Blood culture performed, yes vs. no |
| <0.001 | 6.91 (2.96-16.15) | <0.001 | 8.26 (4.37-15.61) | Urine culture performed, yes vs. no |

N=353, Nagelkerke R^2^=0.414. AGE= Acute gastroenteritis; CI=Confidence interval; Df=Degree of freedom; OR=Odds ratio

**Supplementary Table 7: Demographic and clinical factors associated with unnecessary antibiotic use among** **children hospitalized with laboratory-confirmed RVGE or norovirus AGE — Bivariate analysis**

| **P value** | **No antibiotic treatment**  **N=309** | **Antibiotic treatment**  **N=25** |  |
| --- | --- | --- | --- |
| 0.396 |  |  | Age (months), N (%) |
|  | 118 (38.2%) | 13 (52.0%) | 0-11 |
|  | 129 (41.7%) | 8 (32.0%) | 12-23 |
|  | 62 (20.1%) | 4 (16.0%) | 24-59 |
| 0.630 | 170 (45.0%) | 15 (60.0%) | Sex, males, N (%) |
| 0.059 | 162 (52.4%) | 18 (72.0%) | Ethnicity (Jewish vs. Arabs), N (%) |
| 0.042 |  |  | Residential socioeconomic status rank, N (%) |
|  | 121 (41.4%) | 6 (26.1%) | 1-3 (Low) |
|  | 113 (38.7%) | 15 (65.2%) | 4-5 (Intermediate) |
|  | 58 (19.9%) | 2 (8.7%) | 6-10 (High) |
| 0.570 |  |  | Year of admission, N (%) |
|  | 26 (8.4%) | 0 (0.0%) | 2008 |
|  | 31 (10.0%) | 1 (4.0%) | 2009 |
|  | 108 (35.0%) | 9 (36.0%) | 2010 |
|  | 39 (12.6%) | 4 (16.0%) | 2011 |
|  | 32 (10.4%) | 5 (20.0%) | 2012 |
|  | 53 (17.2%) | 5 (20.0%) | 2013 |
|  | 14 (4.5%) | 1 (4.0%) | 2014 |
|  | 6 (1.9%) | 0 (0.0%) | 2015 |
| <0.001 | 50 (16.2%) | 7 (28.0%) | Background diseases, N (%) |
| 0.070 | 105 (34.0%) | 13 (52.0%) | Previous lifetime hospitalization, N (%) |
| 0.037 | 40 (12.9%) | 7 (28.0%) | Previous AGE hospitalization, N (%) |
| 0.070 | 104 (34.0%) | 13 (52.0%) | Fever on admission, N (%) |
| 0.316 | 297 (96.1%) | 25 (100.0%) | Vomiting, N (%) |
| 0.997 | 217 (73.8%) | 16 (69.6%) | Number of stools on the severe day, N (%) |
|  | 99 (32.0%) | 8 (32.0%) | 0-5 |
|  | 210 (68.0%) | 17 (68.0%) | ≥6 |
| 0.341 | 66 (82.5%) | 7 (70.0%) | Receiving rotavirus vaccine*, N (%) |
| <0.001 | 11 (3.6%) | 8 (32.0%) | Chest-X-ray test performed, N (%) |
| <0.001 | 59 (19.1%) | 12 (48.0%) | Blood culture performed, N (%) |
| <0.001 | 5 (1.6%) | 5 (20.0%) | Urine culture performed, N (%) |
| 0.443 | 11.9 (1.2), N=289 | 11.7 (1.6), N=24 | Hemoglobin (g/dl), mean (SD) ^#^ |
| 0.006 | 390.7 (127.3), N=289 | 467.9 (175.3), N=24 | Platelets (K/µl), mean (SD) ^#^ |
| <0.001 | 11.8 (4.8), N=289 | 15.5 (8.3), N=24 | Leukocytes (K/µl), mean (SD) ^#^ |
| 0.004 | 6.9 (4.2), N=289 | 9. (5.83), N=24 | Neutrophils (K/µl), mean (SD) |
| 0.429 | 3.8 (2.8), N=289 | 4.3 (2.5), N=24 | Lymphocytes(K/µl), mean (SD) ^#^ |
| 0.140 | 79.2 (19.3), N=288 | 85.3 (16.2), N=23 | Glucose (mg/dL), mean (SD) ^#^ |
| 0.437 | 14.6 (6.7), N=290 | 13.4 (6.7), N=23 | Blood urea nitrogen(mg/dL), mean (SD) ^#^ |
| 0.745 | 0.3 (0.1), N=280 | 0.3 (0.1), N=22 | Creatinine(mg/dL), mean (SD) ^#^ |
| 0.009 | 6.4 (11.7), N=273 | 12.7 (16.4), N=22 | C-reactive protein (mg/L), median (IQR) ^#^ |
| 0.470 | 138.8 (4.2), N=288 | 139.4 (4.6), N=23 | Sodium mEq/l, mean (SD) ^#^ |
| 0.048 | 4.5 (0.6), N=264 | 4.8 (1.0), N=22 | Potassium mEq/l, mean (SD) ^#^ |

*This analysis is based on children with viral AGE eligible for rotavirus vaccination in the universal vaccination program – birth cohorts 2011-2015. ^#^ The numbers (N) represent participants with available data. AGE= Acute gastroenteritis; IQR= Interquartile range; SD=Standard deviation.

**Supplementary Table 8: Comparisons in clinical characteristics between children hospitalized with viral AGE in whom a blood culture was performed and those in whom a blood culture was not performed**

| **P value** | **Did not perform blood culture**  **N=931** | **Performed blood-culture**  **N=464** |  |
| --- | --- | --- | --- |
|  |  |  | Symptoms |
| <0.001 | 271 (29.4%) | 243 (52.4%) | Fever on admission, N (%) |
| <0.001 | 833 (89.5%) | 378 (81.5%) | Vomiting, N (%) |
| <0.001 |  |  | Number of stools on the severe day, N (%) |
| <0.001 | 388 (42.0%) | 261 (56.5%) | 0-5 |
|  | 536 (58.0%) | 201 (43.5%) | ≥6 |
|  |  |  | Performing other tests |
| <0.001 | 70 (7.5%) | 76 (16.4%) | Chest-X-ray test performed, N (%) |
| <0.001 | 28 (3.0%) | 56 (12.1%) | Urine culture performed, N (%) |
| 0.005 | 609 (65.4%) | 268 (57.8%) | Rotavirus test performed, N (%) |
| <0.001 |  |  | Rotavirus test results^§^, N (%) |
|  | 226 (37.1%) | 57 (21.3%) | Positive |
|  | 318 (5.2%) | 207 (77.2%) | Negative |
|  | 65 (10.7%) | 4 (1.5%) | Unknown |

^§^ This analysis included only children who performed rotavirus test. AGE= Acute gastroenteritis

**Supplementary Table 9: Comparison in clinical characteristics between children hospitalized with viral AGE who performed chest X-ray imaging and those who did not**

| **P value** | **Did not perform chest X-ray imaging**  **N=1249** | **Performed**  **chest X-ray**  **N= 146** |  |
| --- | --- | --- | --- |
|  |  |  | **Symptoms** |
| <0.001 | 426 (34.4%) | 88 (60.7%) | Fever on admission, N (%) |
| <0.001 | 1104 (88.4%) | 107 (73.3%) | Vomiting, N (%) |
| <0.001 |  |  | Number of stools on the severe day, N (%) |
|  | 561 (45.2%) | 88 (60.7%) | 0-5 |
|  | 680 (54.8%) | 57 (39.3%) | ≥6 |
|  |  |  | **Performed other tests** |
| <0.001 | 61 (4.9%) | 23 (15.8%) | Urine culture performed, N (%) |
| <0.001 | 388 (31.1%) | 76 (52.1%) | Blood culture performed, N (%) |
| 0.013 | 799 (64.0%) | 78 (53.4%) | Rotavirus test performed, N (%) |
| 0.004 |  |  | Rotavirus test results, N (%) |
|  | 270 (33.8%) | 13 (16.7%) | Positive |
|  | 465 (58.2%) | 60 (76.9%) | Negative |
|  | 64 (8.0%) | 5 (6.4%) | Unknown |

AGE= Acute gastroenteritis.

**Supplementary Table 10: Comparison in clinical characteristics between children hospitalized with viral AGE who performed urine culture and those who did not**

| **P value** | **Did not perform a urine culture**  **N=1311** | **Performed urine culture**  **N=84** |  |
| --- | --- | --- | --- |
|  |  |  | Symptoms |
| 0.022 | 473 (36.4%) | 41 (48.8%) | Fever on admission, N (%) |
| <0.001 | 1154 (88.0%) | 57 (67.9%) | Vomiting, N (%) |
| 0.106 |  |  | Number of stools on the severe day, N (%) |
|  | 603 (46.3%) | 46 (55.4%) | 0-5 |
|  | 700 (53.7%) | 37 (44.6%) | ≥6 |
|  |  |  | Performed other tests |
| <0.001 | 123 (9.4%) | 23 (27.4%) | Chest X-ray performed, N (%) |
| <0.001 | 408 (31.1%) | 56 (66.7%) | Blood culture performed, N (%) |
| 0.513 | 827 (63.1%) | 50 (59.5%) | Rotavirus test performed, N (%) |
| 0.010 |  |  | Rotavirus test results^§^, N (%) |
|  | 274 (33.1%) | 9 (18.0%) | Positive |
|  | 485 (58.6%) | 40 (80.0%) | Negative |
|  | 68 (8.2%) | 1 (2.0%) | Unknown |

AGE= Acute gastroenteritis.

**Supplementary Table 11:** **Antibiotic treatment for children hospitalized with viral AGE, while excluding children in whom urine culture or chest X-ray test were performed, N=1188) overall and by timing relative to hospital admission**

| **At discharge** | **During hospitalization** | **Before admission** | **Antibiotics treatment (any)** |  |
| --- | --- | --- | --- | --- |
| **N (%)** | **N (%)** | **N (%)** | **N (%)** |  |
|  |  |  |  | **Received antibiotics** |
| 35/1188 (2.9%) | 87/1188 (7.3%) | 77/1188 (6.5%) | 153/1188 (12.9%) | **Yes** |
| NA | NA | NA | 1035/1188 (87.1%) | **No** |

AGE= Acute gastroenteritis. NA: the percentages were calculated among children who received antibiotics.

**Supplementary Table 12: Logistic regression model of factors associated with unnecessary antibiotic use during and after hospitalizations in children with viral AGE, excluding children in whom a urine culture or chest X-ray test was performed**

| **P value** | **Adjusted OR (95% CI)** | **Variable** |
| --- | --- | --- |
| 0.989 | 0.99 (0.60-1.69) | Sex (males vs. female) |
| 0.064 | 0.31 (0.09-1.07) | Ethnicity (Jewish vs. Arab) |
| 0.708 | 1.01 (0.98-1.03) | Age (months), continuous variable |
| 0.079 | Df=2 | Residential socioeconomic status rank |
|  | Reference | 1-3 (Low) |
| 0.348 | 0.56 (0.17-1.87) | 4-5 (Intermediate) |
| 0.052 | 0.23 (0.05-1.01) | 6-10 (High) |
| 0.246 | 1.39 (0.79-2.41) | Fever at admission, yes vs. no |
| 0.436 | 1.43 (0.58-3.52) | Vomiting, yes vs. no |
| <0.001 | 1.09 (1.05-1.14) | Leukocytes (K/µl), continuous variable |
| <0.001 | 1.03 (1.02-1.04) | C-reactive protein (mg/L), continuous variable |
| <0.001 | 0.19 (0.08-0.42) | Rotavirus vaccination (yes vs. no) |
| 0.094 | 2.38 (0.86-6.57) | Birth cohort (2011-2015 vs. other years) |
| 0.063 | 2.09 (0.96-4.55) | Year of admission 2008-2010 vs. 2011-2015 |
| 0.059 | 1.85 (0.98-3.45) | Blood culture performed, yes vs. no |
| 0.416 | 1.25 (0.73-2.13) | Number of stools ≥ 6 vs. 0-5 |

N=887, Nagelkerke R^2^=0.179. AGE= Acute gastroenteritis; CI=Confidence interval; Df=Degree of freedom; OR=Odds ratio.

**Supplementary Table 13: Antibiotic treatment in children hospitalized with viral AGE excluding children in whom a blood culture, urine culture or chest X-ray test, were performed (N=841) overall and by timing relative to hospital admission**

| **At discharge** | **During hospitalization** | **Before admission** | **Antibiotics treatment (any)** |  |
| --- | --- | --- | --- | --- |
| **N (%)** | **N (%)** | **N (%)** | **N (%)** |  |
|  |  |  |  | **Received antibiotics** |
| 22 (2.6%) | 45 (5.4%) | 52 (6.2%) | 88 (10.5%) | **Yes** |
| NA | NA | NA | 753 (89.5%) | **No** |

AGE= Acute gastroenteritis. NA: the percentages were calculated among children who received antibiotics.

**Supplementary Table 14: Logistic regression model of factors associated with unnecessary antibiotic use during and after hospitalizations in children with viral AGE, while excluding children in whom a blood culture, urine culture, or chest X-ray test was performed**.

| **P value** | **Adjusted OR (95% CI)** | **Variable** |
| --- | --- | --- |
| 0.737 | 1.13 (0.55-2.31) | Sex (males vs. female) |
| 0.902 | 0.93 (0.26-3.28) | Ethnicity (Jewish vs. Arab) |
| 0.866 | 1.01 (0.97-1.03) | Age (months), continuous variable |
| 0.106 | Df=2 | Residential socioeconomic status rank |
|  | Reference | 1-3 (Low) |
| 0.291 | 1.95 (0.56-6.75) | 4-5 (Intermediate) |
| 0.519 | 0.52 (0.09-3.44) | 6-10 (High) |
| 0.663 | 1.19 (0.55-2.58) | Fever at admission, yes vs. no |
| 0.799 | 0.90 (0.26-2.86) | Vomiting, yes vs. no |
| 0.160 | 1.05 (0.98-1.12) | Leukocytes (K/µl), continuous variable |
| <0.001 | 1.03 (1.02-1.04) | C-reactive protein (mg/L), continuous variable |
| 0.017 | 0.23 (0.07-0.77) | Rotavirus vaccination (yes vs. no) |
| 0.267 | 2.13 (0.56-8.04) | Birth cohort (2011-2015 vs. other years) |
| 0.028 | 2.68 (1.11-6.47) | Year of admission 2008-2010 vs. 2011-2015 |
| 0.824 | 1.09 (0.53-2.24) | Number of stools ≥ 6 vs. 0-5 |

N=603, Nagelkerke R^2^=0.140. AGE= Acute gastroenteritis; CI=Confidence interval; Df=Degree of freedom; OR=Odds ratio.
